# Intelligent Defect Detection of Ancient City Walls Based on Computer Vision

**DOI:** 10.3390/s25165042

**Published:** 2025-08-14

**Authors:** Gengpei Zhang, Xiaohan Dou, Leqi Li

**Affiliations:** School of Electronic Information and Electrical Engineering, Yangtze University, Jingzhou 434100, China

**Keywords:** ancient architecture defect detection, image data augmentation, YOLO, 3D reconstruction

## Abstract

As an important tangible carrier of historical and cultural heritage, ancient city walls embody the historical memory of urban development and serve as evidence of engineering evolution. However, due to prolonged exposure to complex natural environments and human activities, they are highly susceptible to various types of defects, such as cracks, missing bricks, salt crystallization, and vegetation erosion. To enhance the capability of cultural heritage conservation, this paper focuses on the ancient city wall of Jingzhou and proposes a multi-stage defect-detection framework based on computer vision technology. The proposed system establishes a processing pipeline that includes image processing, 2D defect detection, depth estimation, and 3D reconstruction. On the processing end, the Restormer and SG-LLIE models are introduced for image deblurring and illumination enhancement, respectively, improving the quality of wall images. The system incorporates the LFS-GAN model to augment defect samples. On the detection end, YOLOv12 is used as the 2D recognition network to detect common defects based on the generated samples. A depth estimation module is employed to assist in the verification of ancient wall defects. Finally, a Gaussian Splatting point-cloud reconstruction method is used to achieve a 3D visual representation of the defects. Experimental results show that the proposed system effectively detects multiple types of defects in ancient city walls, providing both a theoretical foundation and technical support for the intelligent monitoring of cultural heritage.

## 1. Introduction

**Figure 1:**
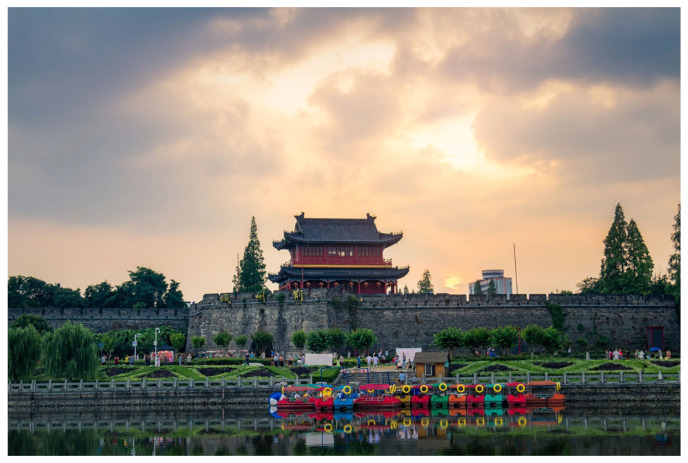
Ancient city wall of Jingzhou.

Ancient city walls (as shown in Figure 1), as significant material relics of historical civilizations, embody the profound imprints of centuries of urban development, military defense, and cultural evolution. They serve as vital cultural links connecting the past with contemporary society. The masonry structures, construction techniques, and spatial layouts not only reflect the advanced architectural technologies of ancient times but also carry rich historical information across dimensions such as geography, politics, and religion. In recent years, many ancient city walls have been increasingly threatened by structural damage and material degradation due to a combination of factors—including weathering erosion [1,2], rainfall erosion [3,4], vegetation intrusion [5,6], and urban development—posing severe challenges to their sustainable preservation.

Inspecting ancient city walls is an effective means of ensuring their sustainable preservation [7,8]. Current methods for detecting structural damage and material deterioration in ancient city walls primarily rely on manual inspections, percussion testing, infrared thermography [9,10,11], and ground-penetrating radar (GPR) [12,13]. Manual inspections depend heavily on subjective expert judgment, are time-consuming and labor-intensive, and struggle to meet the demands of rapid, large-scale site surveys. Percussion testing carries the risk of causing secondary damage to the structure, conflicting with the principle of minimal intervention in cultural heritage preservation. Although infrared thermography and GPR are non-destructive techniques, they also have limitations. Infrared thermography is highly sensitive to environmental conditions—such as temperature, humidity, and sunlight—which can compromise image quality and lead to instability and inaccuracy in detection results. GPR is more suited to point-based inspection and faces challenges in efficiently and continuously surveying large or complex wall structures; furthermore, interpreting its data is complex and requires highly trained operators. Therefore, to achieve the goal of sustainable protection, it is essential to develop more efficient, automated, and environmentally adaptive detection methods for ancient city walls.

Establishing an effective detection system for ancient city walls is of great significance for their sustainable preservation. In recent years, defect-detection technologies based on computer vision have made significant progress. Image processing, as a foundational technique in computer vision, is widely applied across various vision-based tasks. Among these, low-light image enhancement [14,15] has attracted broad research interest, giving rise to numerous solutions aimed at improving visual quality and facilitating downstream vision tasks. Pérez-Zárate et al. [16] proposed ALEN, a dual-path enhancement method that processes uniform and non-uniform low-light images separately, balancing visual quality and performance in subsequent tasks. Generative Adversarial Networks (GANs) have also shown promise in addressing low-light conditions. Wang et al. [17] used GANs to generate high-quality, enhanced images in dark environments, demonstrating the potential of generative models in visual enhancement. Liu et al. [18] constructed a realistic low-light dataset and introduced a conditional diffusion probabilistic model to tackle issues like noise and information loss. Zhang et al. [19] designed the RSCDM (Retinex Self-Conditioned Diffusion Model), which integrates illumination learning to optimize enhancement effects. Their work primarily focused on innovations in diffusion models and datasets. Wang et al. [20] combined image signal processors (ISPs) with convolutional neural networks (CNNs) to achieve efficient real-time enhancement in coal mine Internet of Things (IoT) scenarios. Lastly, Turab’s comprehensive review [21] offered valuable insights into the full landscape of image signal-processing methods, highlighting the ongoing evolution and convergence of traditional and modern techniques in the field of illumination enhancement.

Surface defect and crack detection [22,23,24] has become a mature field within computer vision. In the domain of surface defect detection, Chen et al. (2024) proposed a lightweight model called LGCL-CenterNet to enhance real-time detection efficiency [25]. Similarly, Liu et al. (2024) developed a lightweight machine learning model, incorporating data augmentation techniques to achieve high-accuracy results [26]. Their work emphasized the role of data augmentation in improving model robustness. For crack detection, the literature shows a clear trend of combining advanced deep learning architectures with attention mechanisms. Li et al. (2024) improved the Faster R-CNN model by integrating residual networks and squeeze-and-excitation modules, thereby enhancing feature extraction and focus for crack identification [27]. Wang et al. (2025) developed an automatic labeling and feature fusion method based on the watershed algorithm, enabling efficient crack segmentation with minimal human intervention [28]. This addresses the common bottleneck of labor-intensive dataset annotation in crack detection. The importance of dataset quality and interpretability in defect detection has also been highlighted. Ruzavina et al. (2025) introduced the SteelBlastQC dataset, which includes interpretable neural network models that allow users to intuitively identify defect areas and understand model decisions [29]. Such datasets and models are essential for building trust and transparency in automated detection systems. Furthermore, Li et al. (2025) proposed the GYU-DET dataset for bridge-surface defect detection, demonstrating efforts to improve dataset diversity and labeling accuracy, thus supporting more robust and generalizable detection models [30]. Progress in dedicated detection systems for specific applications has also been notable. Wang et al. (2025) developed a monocular line-scan vision system capable of detecting cracks on highly reflective bearing ball surfaces, showing strong potential for high-precision defect detection in challenging environments [31]. Similarly, Zhao Zhanfang et al. (2025) enhanced the YOLO framework for damage detection on wind turbine blades, achieving significant improvements in mean average precision (mAP) and multi-scale defect recognition in complex conditions [32]. Dou et al. (2024) developed an internal thread image acquisition system combined with a specialized lighting design, employing various image-processing techniques and YOLO for detecting surface defects in internal threads [33]. In summary, computer vision-based techniques for surface defect and crack detection offer clear advantages over the traditional methods used for ancient city wall inspection. These include higher detection accuracy, better resistance to environmental interference, greater automation, and improved efficiency. As such, applying these technologies to ancient wall defect detection is both feasible and promising.

At the same time, point-cloud reconstruction technology—one of the research hotspots in the field of computer vision—has seen significant advancements in its application to defect detection in recent years [34,35]. Arav et al. (2024) [36] proposed a learning-based approach that enables surface anomaly detection by analyzing reconstruction errors in point-cloud models. Liu et al. (2024) [37] extended this paradigm by introducing a unified, unsupervised framework capable of detecting a wide range of defects in products without relying on model priors. Yang et al. (2024) [38] demonstrated that a novel entropy-thresholding method for high-fidelity depth image reconstruction can significantly enhance structural completeness, even under noisy conditions. Similarly, Zhou et al. (2024) [39] focused on multi-view reconstruction and data augmentation techniques to improve the accuracy of point-cloud-based models, thus enabling more reliable anomaly detection in practical settings. Addressing the challenges posed by complex geometries and dynamic environments, Deng et al. (2025) [40] proposed an integrated approach combining computer vision with SLAM (Simultaneous Localization and Mapping) for 3D reconstruction and crack detection in concrete structures. Wei et al. (2025) [41] introduced a semantic consistency inference mechanism that leverages semantic relationships within point clouds to enhance the accuracy of 3D object detection. Chen et al. (2025) [42] developed a point-cloud completion network based on triangular cone features, addressing the limitations of unstructured point prediction. Their approach improves robustness and adaptability, highlighting the importance of reliable reconstruction algorithms, semantic inference, and efficient data processing in point-cloud-based defect detection. Compared with traditional 2D detection methods, defect detection via point-cloud reconstruction supports multi-angle analysis and 3D visualization, providing a powerful data foundation for the spatial assessment of defects. Applying point-cloud reconstruction to ancient city wall inspections not only enables spatial modeling and three-dimensional recognition of wall structures but also supports the creation of digital archives, the monitoring of structural deformations, and virtual restoration. These capabilities bring new possibilities for the digital preservation and scientific management of cultural heritage sites like ancient city walls [43,44,45], demonstrating broad application prospects.

This study addresses four common types of critical defects in ancient city walls—missing bricks, cracks, salt crystallization, and vegetation erosion—by constructing a multi-level, multi-dimensional defect-detection system tailored to their characteristics. On the 2D detection level, the system adopts YOLOv12—one of the latest iterations of the YOLO series—to identify surface defects on ancient city walls. On the 3D-detection level, depth estimation methods are employed to detect and analyze defects with prominent depth features. Additionally, Gaussian Splatting-based point-cloud reconstruction and efficient rendering techniques are utilized to perform 3D modeling of the walls, enabling three-dimensional visualization of defects and providing data support for sustainable conservation. The system is fundamentally data-driven and spatially supported, forming a robust technical foundation for intelligent detection, long-term monitoring, and sustainable protection of ancient city wall deterioration. To enhance the system’s robustness, a series of image-processing modules was incorporated prior to the detection stage. Considering the structural complexity of the subject, an illumination enhancement module was included to mitigate the impact of environmental factors such as shadowing. A deblurring module [46,47] was also added to address dynamic blurring caused by UAV-based image acquisition. Furthermore, a data augmentation module [48,49] was integrated to overcome the limitations posed by the scarcity of defect images, improving YOLOv12’s detection accuracy and ensuring more reliable recognition of various wall defects.

## 2. Theoretical Analysis

**Figure 2:**
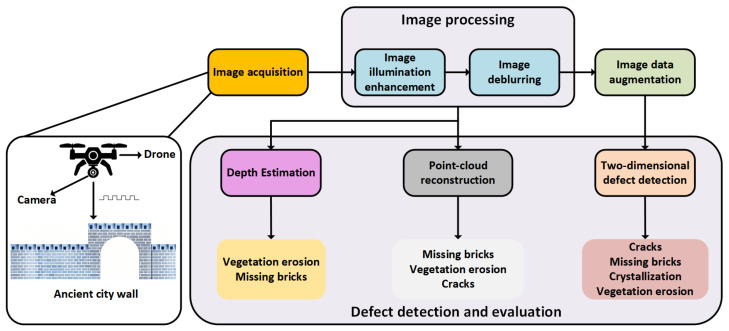
Framework of ancient city wall defect detection.

In the task of defect detection for ancient city walls, this study establishes a comprehensive visual computing workflow. As illustrated in Figure 2, the proposed system follows a primary pipeline consisting of “image acquisition—image processing—2D defect detection—3D modeling”. It encompasses multiple processing stages, including dynamic deblurring, illumination enhancement, data augmentation, object detection, depth estimation, and point-cloud reconstruction.

### 2.1. Image Acquisition

**Figure 3:**
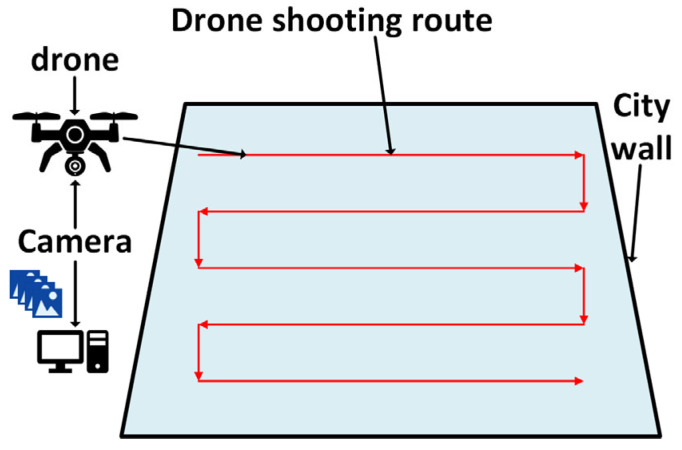
UAV image acquisition path.

In this study, image acquisition was conducted using a UAV (Unmanned Aerial Vehicle) equipped with a camera. Compared with traditional ground-based photography or fixed installations, UAV-based image capture offers a lightweight and highly flexible solution. It allows for multi-angle data collection while significantly reducing manpower and material costs, thereby improving both efficiency and adaptability. As shown in Figure 3, to ensure both flight efficiency and comprehensive image coverage, a horizontal back-and-forth scanning pattern was adopted for the UAV flight path. The flight plan was carefully designed to balance flight range and data coverage, ensuring that high-quality, comprehensive imagery of the ancient city wall structure could be obtained within the constraints of limited flight time and available resources.

**Figure 4:**
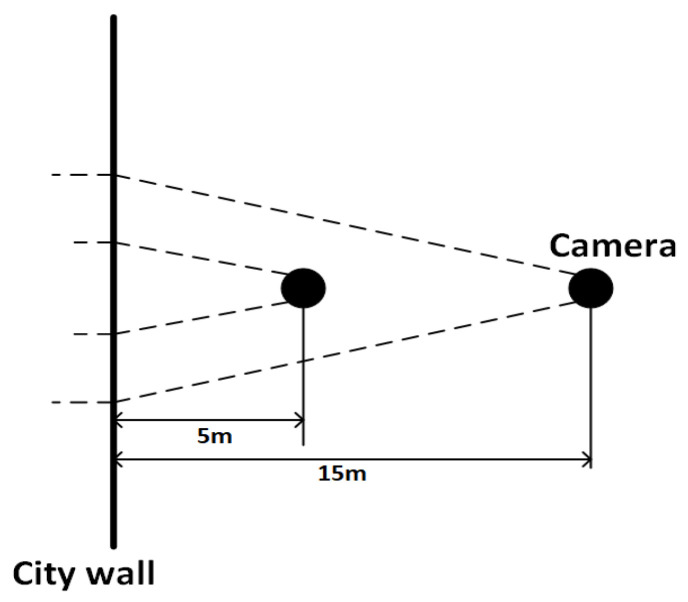
Illustration of the distance between the camera and the ancient city wall.

During the image data acquisition process for the ancient city wall, as illustrated in Figure 4, the UAV employed a multi-view shooting strategy by adjusting its distance from the wall to capture high-quality images for various purposes. To evaluate the imaging effectiveness at different distances, this study utilized a geometric perspective projection model—integrated with actual parameters from the aerial equipment—to estimate image coverage area and resolution.

In this experiment, the average height H of the target is 8.83 m. The vertical field of view angle θ of the captured image is 78°. The UAV is equipped with a camera with an equivalent focal length f of 4.5 mm, and a sensor size of 1/2.3″ (corresponding to a diagonal of 7.7 mm). The image resolution is 4000 × 3000 (i.e., 12 MP). According to the field of view calculation formula for cameras,

(1) $$FOV=2\cdot arctan\left(\frac{d}{2f}\right)$$

where d is the sensor width; f represents the equivalent focal length. Based on actual parameters, the horizontal field of view (FOV) is approximately 69.4°, and the vertical FOV is about 52.1°. At a shooting distance of 5 m, the vertical coverage range is approximately the following:

(2) $${\mathrm{Vertical}\;\mathrm{coverage}}_{5m}=2\cdot 5\cdot tan\left(\frac{52.1^{\circ }}{2}\right)\approx 4.94\;m$$

That is, the image can cover approximately 5 m vertically. Combined with the close-range details of the ancient city wall, this allows for complete visualization of brick joints, cracks, localized salt crystallization, and other fine texture features. Based on a 4000-pixel image, the vertical coverage per pixel is calculated as outlined below:

(3) $${GSD}_{5m}=\frac{4.94\;m}{3000\;px}\approx 1.65\;mm/px$$

Therefore, at a distance of 5 m, the image spatial resolution is approximately 1.65 mm per pixel, which meets the requirements for detecting fine defects and ensures the quality of the experimental data. This is further supported by sample defect size examples presented in the Experiments Section.

At a shooting distance of 15 m, the vertical coverage of the image is as follows:

(4) $${\mathrm{Vertical}\;\mathrm{coverage}}_{15m}=2\cdot 15\cdot tan\left(\frac{52.1^{\circ }}{2}\right)\approx 14.8\;m$$

This coverage range significantly exceeds the average height of the ancient city wall (8.83 m), making it sufficient to capture the entire structure within a single frame. These images can serve as comparative data for evaluating the accuracy of point-cloud reconstruction models.

### 2.2. Image Processing

#### 2.2.1. Image Deblurring

Due to the high-speed movement of UAVs, the captured images often suffer from varying degrees of motion blur, which interferes with subsequent processes such as illuminance enhancement, image data augmentation, 2D defect detection, depth estimation, and 3D reconstruction. Restormer, proposed by Zamir et al. (2022) [50], has demonstrated state-of-the-art performance in image deblurring tasks. With only 26.1M parameters and 52.0 GMACs, it achieves high-quality restoration, showing remarkable texture reconstruction capability and computational efficiency under high-resolution and complex blur conditions.

This study adopts the Restormer model to address the image deblurring problem. Unlike traditional CNN-based approaches [51,52], its core innovation lies in the design of the Transformer module, which includes two key components: Multi-Dconv Head Transposed Attention (MDTA) and Gated-Dconv Feed-Forward Network (GDFN).

**Figure 5:**
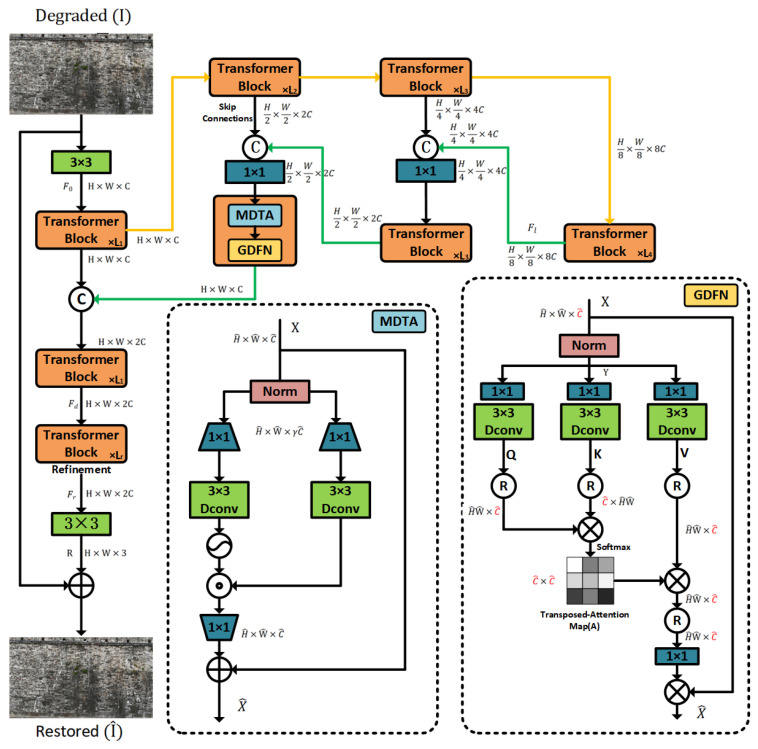
Restormer network flowchart.

The Restormer network process is shown in Figure 5. The MDTA module introduces a cross-channel attention mechanism that shifts attention computation from the spatial domain to the feature domain, reducing the complexity to a linear scale. This is particularly crucial for high-resolution images of ancient city walls captured by UAVs, enabling more efficient modeling of long-range pixel dependencies. The GDFN module enhances information expression and suppresses redundant channels by combining gating mechanisms with depthwise convolutions. It selectively transmits useful features while filtering out irrelevant noise, using two parallel transformation paths to perform feature selection and enhancement—thus further improving the texture restoration quality of the deblurred images.

#### 2.2.2. Illumination Enhancement

Due to limitations such as natural lighting conditions, terrain occlusion, and the surface reflectance of aged materials, UAV-acquired images of ancient city walls often suffer from various degradation issues, including low brightness, poor contrast, and blurred edges. These problems not only impair visual perception but also directly limit the accuracy of subsequent defect-detection algorithms. To address this challenge, image Illumination enhancement [53] has emerged in recent years as a critical area of image preprocessing research, aiming to restore realistic illumination and structural details through algorithmic approaches, thereby improving overall image quality and downstream task performance.

**Figure 6:**
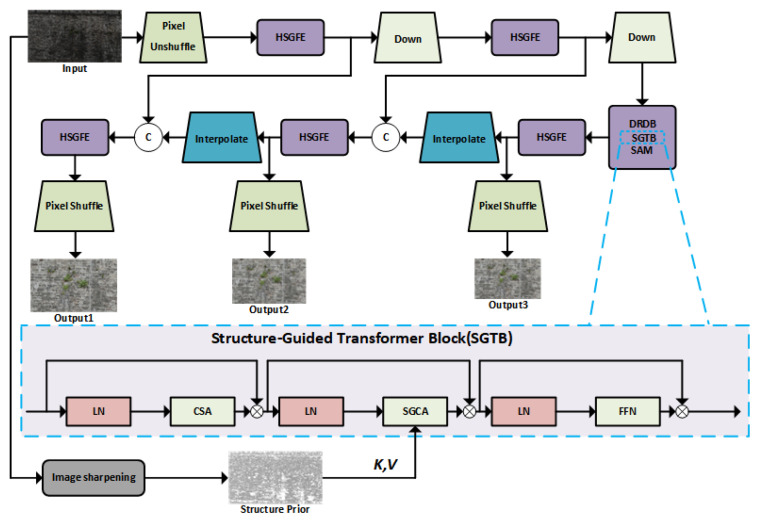
SG-LLIE network flowchart.

The Structure-Guided Low-Light Image Enhancement (SG-LLIE) model, proposed by Dong et al. [54], demonstrates outstanding performance in enhancing low-light images. Architecturally, SG-LLIE adopts a U-Net-style encoder–decoder framework, integrating a multi-scale feature fusion module (SAM) and a hierarchical structure-guided feature extractor (HSGFE). Within each HSGFE layer—in addition to employing dilated residual dense blocks (DRDB) for local feature enhancement—the most critical component is the Structure-Guided Transformer Block (SGTB), which incorporates three key mechanisms: Channel Self-Attention (CSA), Structure-Guided Cross-Attention (SGCA), and Feed-Forward Network (FFN). The SG-LLIE network process is shown in Figure 6.

In this study, the SG-LLIE model is introduced to handle the illumination enhancement task. By leveraging a structure-guided Transformer architecture, it enables precise modeling of local structural information and effective cross-scale feature interaction. This approach significantly improves image brightness and contrast while preserving structural consistency—ensuring better input quality for subsequent defect-detection modules.

#### 2.2.3. Image Data Augmentation

The acquisition of defect images from ancient city walls is limited by cultural heritage protection laws, physical space constraints, and shooting conditions. As a result, the number of available samples is small and class distribution is imbalanced—this presents a core challenge. LFS-GAN is a generative model specifically designed for few-shot scenarios. It can synthesize high-quality and diverse defect images even when only a few real samples are available, which is something traditional GANs (such as DCGAN [55], CycleGAN [56], and StyleGAN [57]) cannot achieve. Therefore, in this paper, Lifelong Few-Shot Generative Adversarial Network (LFS-GAN) [58] is introduced as the image data augmentation module.

**Figure 7:**
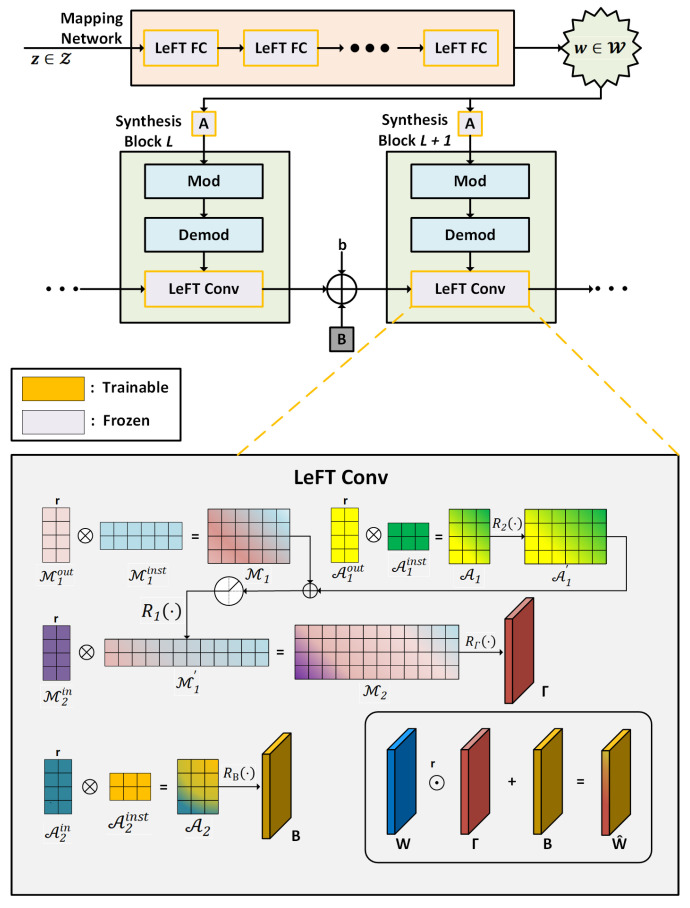
Architecture of LFS-GAN and LeFT factorized modulation.

LFS-GAN introduces a Learnable Factorized Tensor (LeFT), a parameter-efficient factorization module. The core idea is to freeze the backbone weights of the pretrained model and train only the task-specific modulation parameters. This significantly reduces the memory and computational overhead required for parameter updates and helps avoid overfitting. The LFS-GAN structure and the LeFT factorization mechanism are shown in Figure 7.

For a convolutional weight tensor $W\in R^{C_{out}\times C_{in}\times k\times k}$, new weights are generated through the following modulation function:

(5) $$\hat{W}=W⊙\Gamma +B$$

where $\hat{W}$ denotes the new weight after modulation; ⊙ represents the element-wise (Hadamard) product; Γ is the multiplicative modulation factor, used to control the amplitude variation in each parameter in the convolution kernel; B is the additive modulation factor, used to introduce structural bias. Both Γ and B are learnable parameters. The expression W⊙Γ can be interpreted as reweighting the original parameters to emphasize or suppress certain channels. The additive term B provides the ability to model new features. This decoupled modulation mechanism not only significantly reduces the number of parameters and computational resources required for training but also effectively retains the knowledge transfer capability of the original model, avoiding overfitting issues in few-shot learning scenarios.

LFS-GAN also introduces an important loss function—cluster-wise mode seeking loss—designed to enhance the diversity of the generated images. This loss is defined based on the variation rates of the input noise $z_{i}$, intermediate representation $w_{i}$, feature map $F_{l,i}$, and the final output image $I_{i}$, as follows:

(6) $$L_{cms}=min\left(\frac{1}{B}\sum_{i=1}^{B}\left(\frac{\Delta w_{i}}{\Delta z_{i}}+\frac{1}{L}\sum_{l=1}^{L}\left(\frac{\Delta F_{l,i}}{\Delta w_{i}}+\frac{\Delta I_{i}}{\Delta w_{i}}\right)\right)\right)$$

The design of this loss function is centered on encouraging the generation of images that form multiple clusters in the latent space, thereby enhancing the diversity of generated images. Specifically, the formula contains the following two main parts:

1. $\frac{\Delta w_{i}}{\Delta z_{i}}$ measures the influence of the input noise $z_{i}$ on the change in the latent space variable $w_{i}$, ensuring the model’s ability to respond to different variations in the latent space.
2. $\frac{\Delta F_{l,i}}{\Delta w_{i}}+\frac{\Delta I_{i}}{\Delta w_{i}}$, respectively, depict the sensitivity of image features and pixel-level outputs to changes in the intermediate representation. Larger values mean that small perturbations in the latent variables lead to significant image differences, which help to increase the diversity of the generated results.

Moreover, the min operation ensures that the training process focuses on the sample paths with the least variation, effectively mitigating the common-mode collapse problem under few-shot conditions [59]. Ultimately, this loss function guarantees the diversity of synthesized ancient city wall images under various conditions, such as salt crystallization features, missing bricks, and vegetation erosion.

### 2.3. Defect Detection

To achieve comprehensive identification of defects in ancient city walls, this study designs a hierarchical and progressively structured defect-detection system. The target applications of each detection module are summarized in Table 1.

First, by integrating a lightweight YOLOv12-based object detection module, the system can perform preliminary identification of four common types of defects in ancient city walls: missing bricks, cracks, salt crystallization, and vegetation erosion. Given that missing bricks and vegetation erosion exhibit distinct depth characteristics—and considering the limited number of samples for these two defect types—introducing a depth estimation module is especially suitable. In this system, depth estimation serves as an auxiliary verification tool specifically for these two categories. The inclusion of the depth estimation module complements the object detection module by providing a spatial validation of defects.

While numerous studies have reported the application of depth estimation in general object detection and structural health monitoring [60,61,62], its use in ancient wall defect detection remains relatively rare, offering a novel direction for this field.

Finally, from the perspective of sustainable preservation, different defect types exhibit varying spatial structures. Point-cloud data can capture the three-dimensional shape and spatial position of these defects from multiple angles and scales, enabling more accurate differentiation and quantification. Moreover, point-cloud reconstruction is not only a detection technique but also a versatile data presentation method that supports downstream tasks such as digital archiving, historical tracking, restoration assistance, and virtual simulation; thus, it provides strong technical support for the sustainable preservation of ancient city walls.

In summary, the object detection module serves as the foundation for defect identification, the depth estimation module provides auxiliary verification, and the point-cloud reconstruction module functions as both a detection method and an extended technology for heritage conservation. The collaborative integration of these components forms the core of the proposed hierarchical detection and preservation framework for ancient city wall defects.

#### 2.3.1. Two-Dimensional Defect Detection

**Figure 8:**
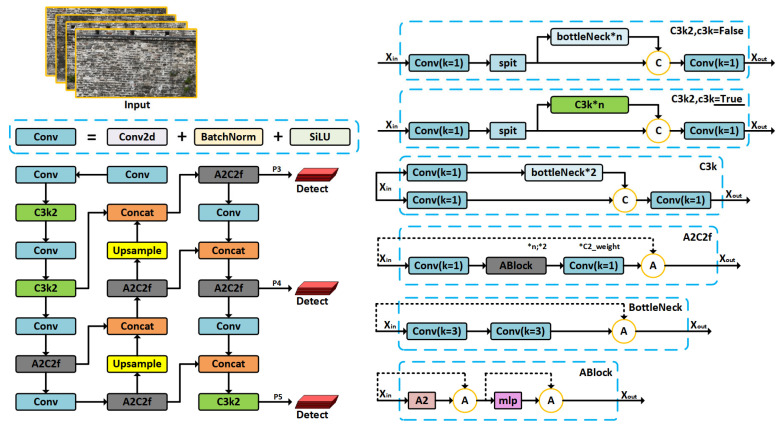
Overall network architecture and key modules of YOLOv12.

YOLOv12 [63] is adopted as the core detection backbone due to its lightweight architecture, high accuracy, and strong adaptability to complex texture backgrounds, making it particularly well-suited for detecting multi-scale surface defects on ancient city walls. Figure 8 illustrates the overall architecture of YOLOv12, including the backbone feature extraction network, feature fusion path, and multi-scale detection heads. YOLOv12 follows the efficient single-stage detection design of the YOLO [64] series. The input consists of sequences of ancient wall images, which are processed through a backbone network to extract multi-layer convolutional features. The feature extraction pipeline includes the C3k2 and A2C2f modules, where C3k2 is a multi-branch residual structure and A2C2f integrates attention mechanisms to enhance the preservation of texture details and responsiveness to high-frequency information. The convolutional building blocks consist of Conv2d, BatchNorm, and SiLU activation functions, which ensure stable convergence while enabling non-linear representation capability.

YOLOv12 employs bottom-up upsampling combined with cross-layer feature concatenation (Concat + Upsample), enabling the integration of fine-grained low-level details and rich high-level semantics for improved detection performance. This generates detection feature maps at three different receptive field scales, allowing the network to handle defects of varying sizes and irregular distribution on ancient city walls.

To further enhance perception, several structural enhancement modules are integrated. The C3k series modules combine residual branches with different channel proportions, improving multi-scale texture awareness while maintaining model compactness. The A2C2f module incorporates the Ablock attention structure, combining the A2 channel attention mechanism and an MLP modeling path. This enables spatial response enhancement to be converted into channel-wise weighted fusion within the feature maps, thereby improving the model’s robustness in defect-detection tasks.

In this study, the lightweight version YOLOv12-S was adopted as the primary detection network. Compared with larger variants such as YOLOv12-L or YOLOv12-X, YOLOv12-S maintains a high detection accuracy while significantly reducing computational complexity and parameter size, making it more suitable for on-board deployment on UAV platforms and large-scale inspection tasks of ancient city walls. In the backbone feature extraction stage, YOLOv12-S employs fewer stacked C3k2 modules and applies a channel pruning strategy to reduce the number of feature channels. This design achieves a balance between detection speed and accuracy while preserving the ability to detect multi-scale defects. Such a configuration effectively meets the requirements of detecting diverse defect types under complex texture backgrounds.

#### 2.3.2. Depth Estimation

In defect-detection systems for ancient city walls, 2D images often fail to fully capture structural variations on wall surfaces. The introduction of depth estimation technology [65] enhances the system’s spatial perception capabilities and enables 3D structural modeling of defect regions.

ZoeDepth [66] has demonstrated excellent performance in zero-shot depth estimation tasks by effectively combining the advantages of relative and metric depth. Through a unified distillation framework and a multi-scale fusion strategy, ZoeDepth addresses the limitations of traditional methods in terms of structural preservation and scale awareness, providing a high-performance, generalizable solution for depth estimation.

This paper incorporates the ZoeDepth depth estimation framework to predict depth in areas affected by missing bricks and vegetation erosion, serving as auxiliary validation for detected defects. ZoeDepth maps input images into pixel-wise depth prediction maps and introduces a Local Planar Guidance (LPG) mechanism to enable precise modeling of structural boundaries.

**Figure 9:**
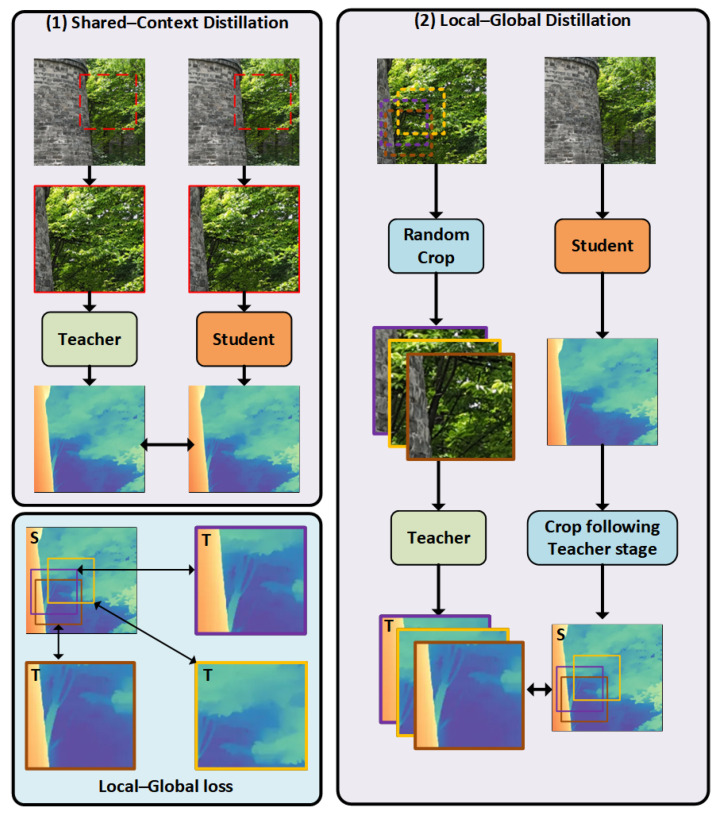
Local–global knowledge distillation mechanism in ZoeDepth.

As shown in Figure 9, the network architecture of ZoeDepth consists of three main components: a backbone encoder, a cross-scale context fusion module, and a depth regression branch. The parallel design of these components allows the model to efficiently capture the multi-scale spatial context, making it particularly suitable for complex scenes involving multi-scale features like missing bricks and vegetation erosion on ancient walls.

#### 2.3.3. Three-Dimensional Reconstruction

To enhance the spatial modeling capabilities of the system for deep structural defects in ancient city walls, this study introduces Gaussian Splatting [67] as the method for 3D reconstruction and efficient rendering. This approach overcomes the limitations of traditional hardware-dependent methods such as structured light [68,69,70] and LiDAR [71,72], offering advantages in terms of lightness, high precision, and real-world scale mapping. It is particularly suitable for non-contact 3D reconstruction tasks in complex historical heritage sites.

Gaussian Splatting is a voxel-based illumination modeling and spatial density regression method. It represents a 3D scene as a collection of transparent particles modeled with anisotropic Gaussian kernels. This structure allows the reconstructed particles to comprehensively express spatial distribution, lighting variation, and surface detail.

During the training phase, multi-view images are processed using a Structure-from-Motion (SfM) algorithm to obtain a sparse 3D reconstruction and initialize camera parameters. Each image frame is then projected onto the initial point cloud to form an initial set of Gaussian particles. The reconstruction is optimized jointly based on reprojection error, color consistency loss, and opacity regularization. The objective function is defined as follows:

(7) $$L_{total}=\lambda_{1}L_{photo}+\lambda_{2}L_{opacity}+\lambda_{3}L_{density}$$

where $L_{photo}$ is the photometric reprojection loss, which ensures color consistency across views; $L_{opacity}$ is the opacity regularization term, which limits particle transparency to avoid excessive overlap; $L_{density}$ adjusts the spatial sparsity of particles. Together, these three components jointly ensure reconstruction accuracy and efficiency.

**Figure 10:**
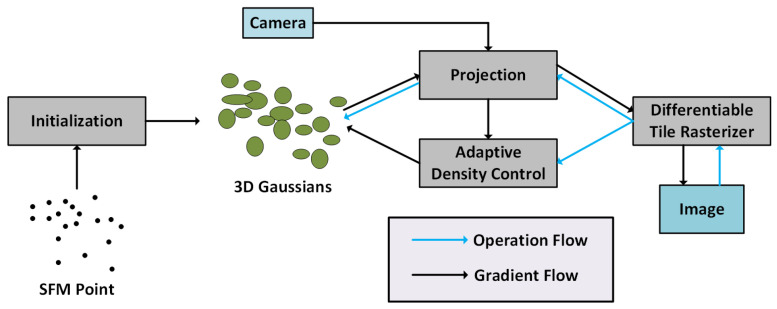
Framework of Gaussian Splatting-based 3D reconstruction.

As illustrated in Figure 10, the core workflow of Gaussian Splatting-based 3D reconstruction begins by initializing the sparse 3D points obtained from Structure-from-Motion (SfM) as Gaussian particles. These particles are then projected onto the image plane based on the corresponding camera poses.

A differentiable tile-based rasterization module is used to render images from the Gaussian particles, and an adaptive density control module is applied to iteratively optimize the Gaussian parameters. This results in high-fidelity 3D reconstruction with accurate spatial and photometric consistency.

## 3. Experiments

### 3.1. Image Processing

**Figure 11:**
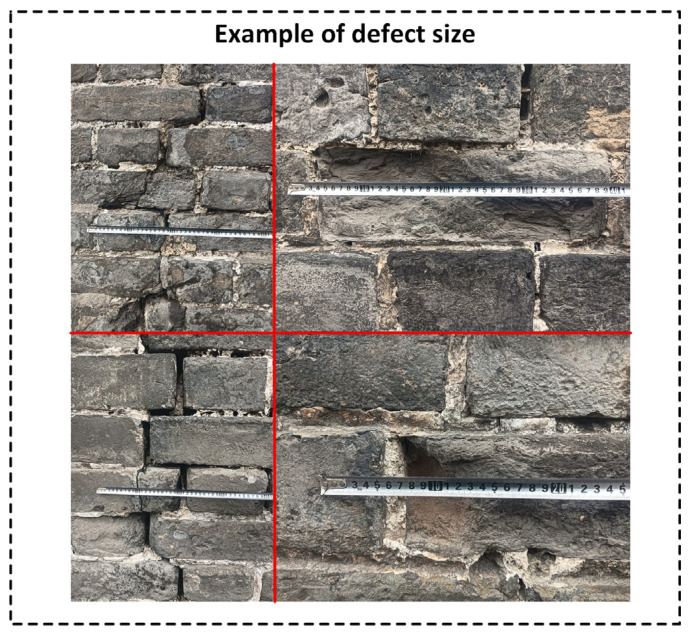
Example of defect size.

In this experiment, Jingzhou Ancient City was selected as the research subject, with the data collection focused on the Binyang Tower section of the city wall, which spans approximately 50 m in length. Layered flight paths were established along the vertical direction of the wall, with a flight height of 5 m from the wall surface. This setup ensured the capture of high-resolution images suitable for identifying defects, thus providing high-quality input for the image processing and detection modules. Based on the calculations in Section 2.1 (Image Acquisition), when the UAV maintains a distance of 5 m from the wall, the vertical image resolution reaches approximately 1.65 mm/pixel, which is sufficient for clearly identifying defects larger than 3.3 mm. As shown in Figure 11, the actual defects on the wall are on the centimeter scale—well above this threshold—confirming that the collected data fully meet the accuracy requirements for subsequent defect detection.

**Figure 12:**
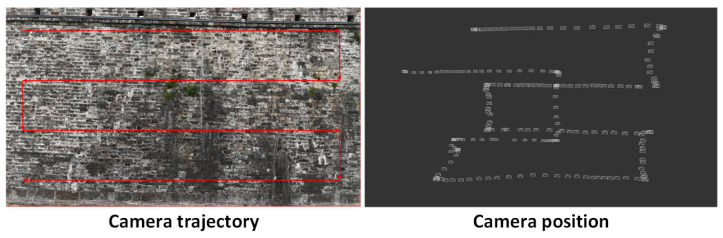
Data acquisition route for the ancient city wall.

The flight altitude was also set at 15 m from the wall surface to ensure the captured images encompass the full view of the ancient city wall. These images serve as comparative data for evaluating the accuracy of the point-cloud reconstruction model. An example of the camera-acquired imagery is shown in Figure 12.

In this experiment, the 50 m data acquisition section of the Binyang Tower area of Jingzhou Ancient City Wall was divided into multiple sub-segments for targeted UAV data collection. This segmented strategy effectively reduced the capture of irrelevant wall areas and enabled the purposeful acquisition of defect-rich regions. Taking the defect of missing bricks as an example, the process was as follows: first, sub-segments with a high concentration or large proportion of missing-brick defects were manually identified. Two representative segments were selected, as follows: (i) a 10 m section on the inner eastern wall; and (ii) a 7 m section on the inner southern wall. Then, based on the planned flight path and to ensure stable camera operation, the UAV flight speed was set to approximately 1 m/s. Finally, each target sub-segment was scanned independently, and the collected data were categorized according to defect type.

A total of seven sub-segments were captured in this experiment, with partial overlaps to guarantee full coverage of the 50 m wall section. After consolidating all collected data, a multi-stage quality control procedure was applied to remove frames with significant vibration, occlusions, or invalid content, retaining only high-quality segments for dataset construction. The effective video length after filtering was 3 min 11 s (191 s). Frames were extracted at a rate of approximately 1.15 frames per second, resulting in a dataset of 220 images, including 50 with missing-brick defects, 100 with cracks, 30 with vegetation erosion, and 40 with salt crystallization.

**Figure 13:**
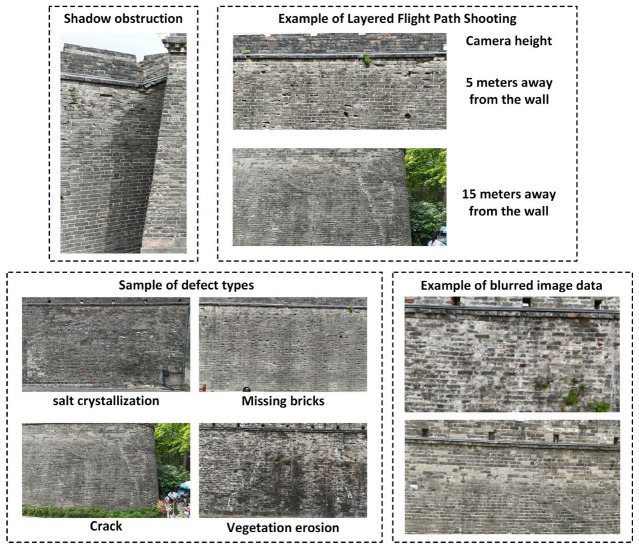
Data collection of the ancient city wall of Jingzhou—examples of various types, as well as errors that occurred.

Although the dataset comprehensively covered four major defect categories, the actual defect distribution in the Binyang Tower section was uneven, with relatively few samples for missing bricks, salt crystallization, and vegetation erosion and a higher frequency of cracks. Vegetation erosion was particularly localized, leading to few valid frames containing this defect type after frame extraction, which caused data scarcity in certain categories and affected the balance of object detection training. This limitation highlights the necessity of the data augmentation module in our framework. In addition, due to the structural characteristics of the city wall (closed square layout with external brick masonry and internal rammed earth) and natural lighting conditions, the north-facing wall had poor illumination. Consequently, 20 extracted frames exhibited insufficient brightness, necessitating the use of the illumination enhancement module. The experiments were conducted under stable weather conditions (moderate temperature, low wind speed, and no precipitation or extreme weather), ensuring UAV flight safety and stable data collection; however, nine frames were still affected by motion blur caused by UAV movement or frame extraction artifacts. Figure 13 presents representative examples of the acquired data.

### 3.2. Image Processing

#### 3.2.1. Image Deblurring

**Figure 14:**
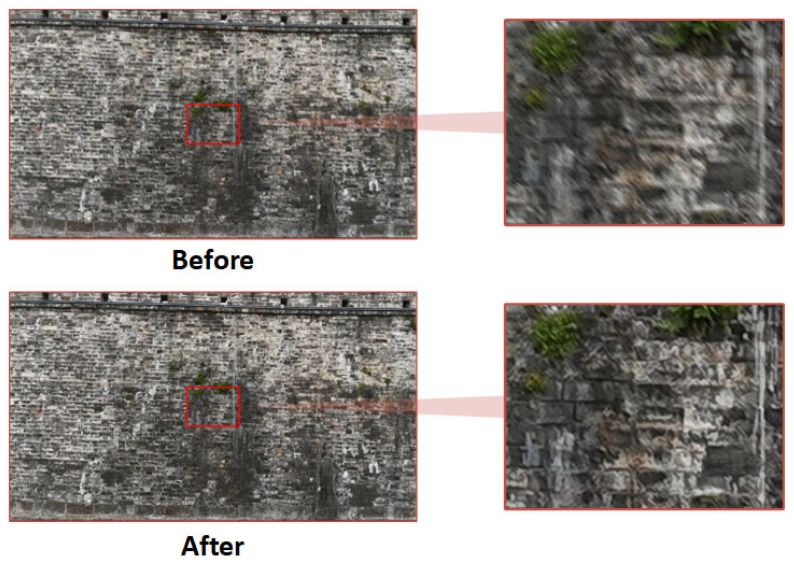
Visual comparison of image deblurring.

The results of image deblurring using the Restormer model are shown in Figure 14. In the original images—limited by oblique shooting angles and insufficient natural lighting—fine edge details were unclear, and the boundaries of bricks and weathered areas were barely distinguishable. After processing with Restormer, the overall brightness became uniform, and previously blurred features—such as cracks in the wall and the edges between bricks—were clearly restored.

Notably, traces of salt crystallization in the lower central area and moss growth in the middle of the wall became clearly identifiable in the enhanced image.

A quantitative analysis was conducted to compare the dynamically deblurred images with the original ones, as shown in Table 2. The Laplacian Variance—an indicator of image sharpness—increased from 1191.62 to 2225.72, representing an 86.8% improvement. The mean edge gradient rose from 101.81 to 142.26, an increase of 39.7%. The Structural Similarity Index (SSIM) reached 0.834, and the Peak Signal-to-Noise Ratio (PSNR) was 24.66 dB.

These results demonstrate that the Restormer model is both robust and effective in handling motion blur in ancient city wall images. It significantly enhances the perceptibility of cracks in walls and brick joints from a subjective visual perspective, while also achieving substantial improvements in objective evaluation metrics. This provides a reliable data foundation for downstream tasks such as image data augmentation, 2D defect detection, depth estimation, and 3D reconstruction.

#### 3.2.2. Illumination Enhancement

**Figure 15:**
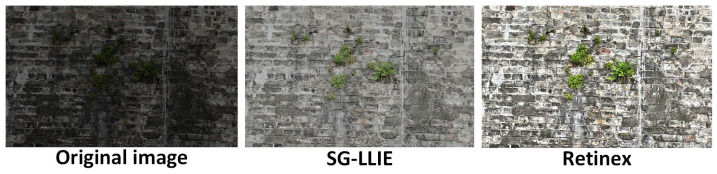
Comparison of illumination enhancement results.

In this experiment, the performance of the SG-LLIE model in enhancing illumination in actual images of ancient city walls was evaluated. A quantitative and qualitative comparison was conducted between SG-LLIE and the Retinex model on the illumination enhancement task. As shown in Figure 15, the original image was captured by a UAV under low-light conditions, resulting in underexposure, blurred structural details, and difficulty in distinguishing between the brick areas and vegetation.

After enhancement by SG-LLIE, the image brightness was significantly improved, and fine textures were clearly reconstructed. Notably, in regions where green vegetation intersects with brick joints, the enhanced image successfully restored multiple previously obscured structural details. Although the Retinex enhanced image also exhibited higher overall brightness, it suffered from issues such as local overexposure, texture blurring, and color saturation imbalance—resulting in unclear boundaries of the actual structural features.

To assess the practical performance of the illumination enhancement module, a quantitative evaluation was carried out between the Retinex model [73] and the structure-guided SG-LLIE proposed in this study. The evaluation included four key metrics: Peak Signal-to-Noise Ratio (PSNR), Structural Similarity Index (SSIM), Learned Perceptual Image Patch Similarity (LPIPS), and No-Reference Image Quality Evaluation (NIQE).

As shown in Table 3, SG-LLIE outperforms Retinex in three key metrics. The PSNR increased from 22.408 to 25.898—a 15.6% improvement—SSIM rose from 0.650 to 0.851, and LPIPS decreased from 0.2 02 to 0.127, indicating better perceptual similarity; however, Retinex achieved a better score in NIQE, with a value of 4.786 compared to 11.828 for SG-LLIE.

Since NIQE is a no-reference metric that is more sensitive to global brightness and contrast than to structural consistency, it may not fully reflect performance in structural-defect-focused heritage scenarios. In such contexts, preserving texture consistency and defect boundary integrity is of higher priority, making SG-LLIE more suitable for ancient wall image enhancement tasks.

Therefore, in the ancient city wall defect-detection system, the enhancement results produced by SG-LLIE are more suitable to serve as the core component for illumination enhancement.

#### 3.2.3. Image Data Augmentation

The key advantage of LFS-GAN lies in its proposed Learnable Factorized Tensor (LeFT) modulation mechanism. This design allows the backbone parameters of the base generative model to remain frozen, while only a low-rank modulation matrix is trained to adapt to new category tasks. This enables few-shot image synthesis while avoiding catastrophic forgetting.

**Figure 16:**
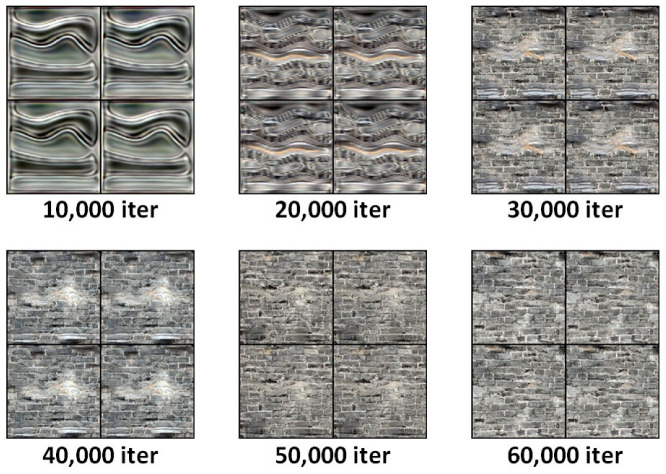
Evolution of synthesized image quality across different training stages of LFS-GAN.

As shown in Figure 16, the figure presents typical synthesized samples from the LFS-GAN model at different stages—from random initialization to stable training. In the early stages, the generated images exhibit blurred edges and incomplete defect structures. As training progresses, the connectivity of cracks, the clarity of brick boundaries, and the brightness distribution in salt crystallization areas increasingly resemble real images. By around 60,000 training iterations, the quality of synthesized images approaches that of real samples.

### 3.3. Defect Detection

#### 3.3.1. Two-Dimensional Surface Detection

**Figure 17:**
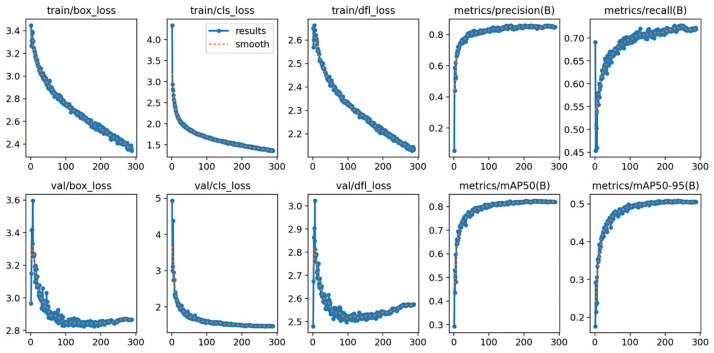
Training loss and detection metric curves for the YOLOv12 model.

The evolution of key performance metrics during the training of the YOLOv12 object detection model is illustrated in Figure 17, including the loss curves for both the training and validation phases, as well as the trends of detection metrics across 300 epochs.

As shown in the training and validation loss curves, the box localization loss, classification loss, and distribution aggregation loss all exhibit a stable downward trend. The most significant decrease occurs within the first 100 epochs, indicating that the constructed dataset and annotation strategy possess strong discriminative power and effectively guide the model in learning to locate and classify defect regions.

Precision rises rapidly in the early epochs and stabilizes around 0.90. Recall surpasses 0.85 after 200 epochs, demonstrating that the model maintains high detection accuracy and a low miss rate in practical scenarios. The model ultimately achieves a mean average precision at the IoU threshold 0.5 (mAP@50) exceeding 0.93, and even under the stricter mAP@50–95 metric, it reaches approximately 0.73. This shows that YOLOv12 performs well not only in detecting simple objects but also under rigorous evaluation criteria involving variable object scales, confidence thresholds, and IoU thresholds.

In summary, the performance metrics validate the effectiveness of training the YOLOv12 model with data generated using LFS-GAN.

#### 3.3.2. Two-Dimensional Depth Estimation

**Figure 18:**
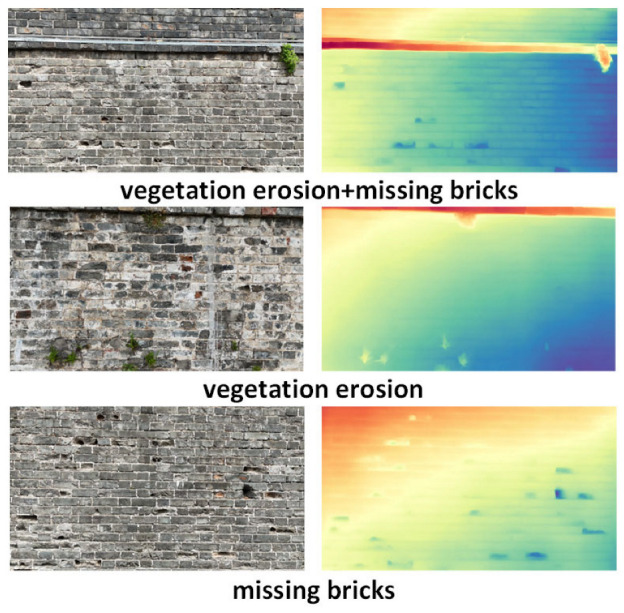
Visualization of defect detection via depth estimation.

As shown in Figure 18, three representative sample groups were selected. Each group includes an RGB image and its corresponding depth heatmap, where colors range from red (closer) to blue (farther) to represent relative depth.

In the “vegetation erosion + missing bricks” sample, the corresponding depth heatmap clearly highlights the raised structural areas on the top and upper-right portion of the wall where vegetation is concentrated, shown as prominent red and yellow high-response zones. This indicates that the model successfully identifies the spatial occlusion caused by vegetation. Meanwhile, several sunken blue patches are observed from the middle to lower sections of the wall, corresponding to large areas of missing bricks in the RGB image. These low-depth values accurately reflect wall surface recession caused by brick loss.

In the “vegetation erosion” sample, the model precisely captures a sharply raised response at a single vegetation point on the top of the wall, forming a consistent red peak in the depth heatmap. The rest of the wall displays a smooth and continuous gradient in mid-to-dark tones, with no abnormal indentations—confirming the absence of other defects.

The “missing bricks” sample shows multiple dark blue recessed regions, with their positions closely matching the visible brick loss in the RGB image. Particularly in the central-right brick joint area, the depth heatmap presents discontinuous bands, simulating the spatial depressions caused by missing bricks.

Overall, the depth estimation module effectively provides spatial verification for certain types of planar defects, reinforcing the reliability of surface-level detection and enhancing the robustness of the defect-detection system.

#### 3.3.3. Three-Dimensional Reconstruction

**Figure 19:**
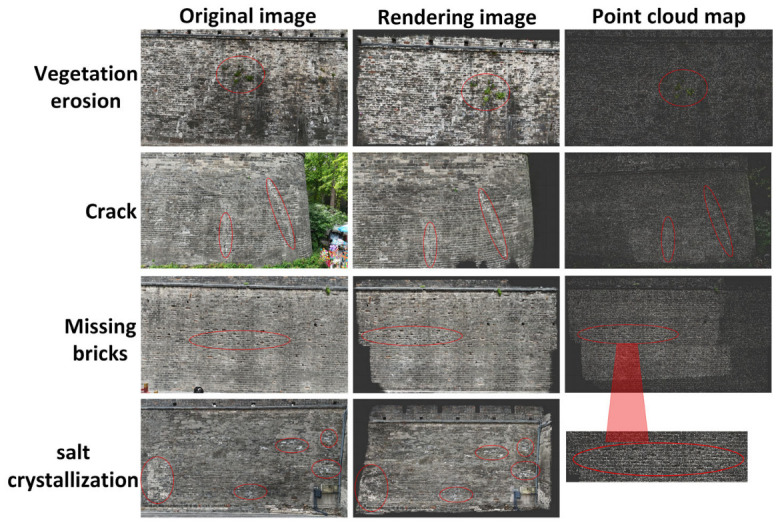
Three-dimensional reconstruction results of Gaussian Splatting.

As shown in Figure 19, the Gaussian Splatting-based reconstruction successfully preserves both the overall structural outline of the wall and the spatial details of brick textures and defect areas. Multiple depressions caused by missing bricks are clearly manifested as high-contrast deformations in the reconstructed view.

Notably, vegetation erosion, missing bricks, and cracks exhibit distinctive features in both the point-cloud and the rendered view, while salt crystallization is visually identifiable mainly in the rendered output. Due to the nature of salt crystallization as a form of surface weathering, it often appears as minor geometric undulations or color deviations in the 3D model. Its relatively weak depth representation makes it difficult to capture clearly in conventional reconstructions, which may hinder accurate defect identification and quantitative analysis.

Vegetation Erosion: In the point cloud, the green vegetation area contrasts noticeably in density with the surrounding wall surfaces. In the rendered image, the location and shape of vegetation attachment are clearly visible, indicating the strong capability of the system to detect and represent raised surface features.

Cracks: Cracks appear as fine linear density variations in the point cloud, and their contours are continuous and sharp in the rendered view. The longitudinal crack textures are faithfully reconstructed, demonstrating the system’s high-resolution capability in capturing fine-grained damage.

Missing Bricks: The point cloud shows multiple uneven-density zones that correspond to depressions caused by brick loss. In the rendered image, these areas are represented by prominent shadows and depth variation, closely matching the original structural geometry.

Salt Crystallization: In the rendered view, parts of the wall exhibit a slightly whitish tone, consistent with the visual manifestation of brick surface degradation caused by salt efflorescence.

## 4. Discussion

### 4.1. Image Processing

To evaluate the individual contributions and synergistic effects of each image-processing submodule within the ancient city wall defect-detection system, this study conducted an ablation experiment at the module level. Five configurations were compared: (1) no image processing; (2–4) applying only one module (deblurring, illumination enhancement, or data augmentation); and (5) applying all three modules together. Performance was assessed using key metrics including mAP, precision, and recall. The experimental results are presented in Table 4.

To further explore the impact of each image-processing submodule on system performance—both independently and in combination—quantitative analysis and extended discussion were conducted. In terms of mAP improvement:

1. Introducing the Restormer deblurring module increased mAP from the baseline 0.751 to 0.804, a gain of 0.053.
2. SG-LLIE illumination enhancement raised it to 0.792 (+0.041).
3. LFS-GAN data augmentation led to an increase to 0.811 (+0.060). When all three modules were used jointly, the system achieved an mAP of 0.839—an overall improvement of 0.088 over the baseline.

To quantify each module’s contribution in the combined configuration, the share of mAP gain relative to the total 0.088 improvement was calculated as follows: LFS-GAN: 68.2%, Restormer: 60.2%, and SG-LLIE: 46.6%. Though the total exceeds 100%, this reflects the non-linear synergistic effect of multi-module integration, indicating that the modules are complementary rather than simply additive.

Analysis of the precision and recall further highlights the distinctive strengths of each module:

1. Restormer contributed most to improving precision (+0.094), showing its strength in sharpening edge clarity and reducing false positives;
2. SG-LLIE enhanced both precision and recall by improving image brightness and structural consistency, aiding the model in identifying defects under low-light conditions;
3. LFS-GAN had the most notable effect on recall (+0.039), demonstrating that generating diverse synthetic samples effectively expands the training set’s boundary and helps the model better recognize underrepresented defect types, thus reducing missed detections.

From a module adaptability perspective:

1. Restormer is particularly suited to mitigating motion blur common in UAV imagery, especially beneficial for detecting cracks and missing bricks along detailed edges;
2. SG-LLIE effectively enhances brightness and texture under challenging lighting conditions such as backlighting, shadow, or obstruction, helping restore the visibility of brick and stone structures;
3. LFS-GAN addresses the limitations of insufficient and imbalanced samples, offering YOLOv12 a high-diversity training dataset—crucial for boosting model robustness.

More importantly, the combined use of all three modules leads to performance improvements not only in key metrics but also through the joint optimization of input image quality, sample distribution, and detection stability. This three-dimensional enhancement pathway—from clarity (Restormer), to structural consistency (SG-LLIE), to dataset balance (LFS-GAN)—builds a robust, adaptive, and high-performance input pipeline for ancient city wall defect detection. It strengthens the system’s ability to handle various defect types, complex lighting environments, and small-sample challenges.

In summary, the ablation experiments confirm both the individual effectiveness and complementary roles of each submodule. The integration strategy of the image-processing modules offers a practical and replicable pathway for front-end design in cultural-heritage-oriented object detection systems, providing valuable insights for similar visual perception and enhancement tasks.

The LFS-GAN model was trained using an NVIDIA RTX 4090 environment, which provided sufficient hardware resources to support efficient training and multi-scale image generation. The detailed training configuration is listed in Table 5.

In this experiment, LFS-GAN was introduced as the core architecture for image generation. A detailed analysis of its training process on a complex-texture dataset was conducted to explore the evolution of its generative performance.

To validate the rationality of the data augmentation module, we conducted a detailed statistical analysis of the original training samples for each defect category. Specifically, the number of valid images for missing bricks, cracks, vegetation erosion, and salt crystallization was only 50, 100, 30, and 40, respectively. The overall dataset was limited in size and exhibited significant class imbalance, making it insufficient for deep detection networks to fully learn multi-scale defect features. To address the issues of data scarcity and imbalance, we employed a Lightweight Feature Separation GAN (LFS-GAN) to independently generate approximately 500 high-diversity synthetic images for each defect type, increasing the total number of samples per class to around 550 and achieving a data expansion ratio of about 10×. This augmentation scale was determined based on multiple experimental convergence tests and prior research experience on small-sample GAN-based augmentation, ensuring enhanced data diversity while avoiding biases caused by an excessively high synthetic-to-real data ratio. The entire generation process took approximately 19 h to complete on a single RTX 4090 GPU, providing a significantly expanded and more balanced dataset for subsequent model training.

**Figure 20:**
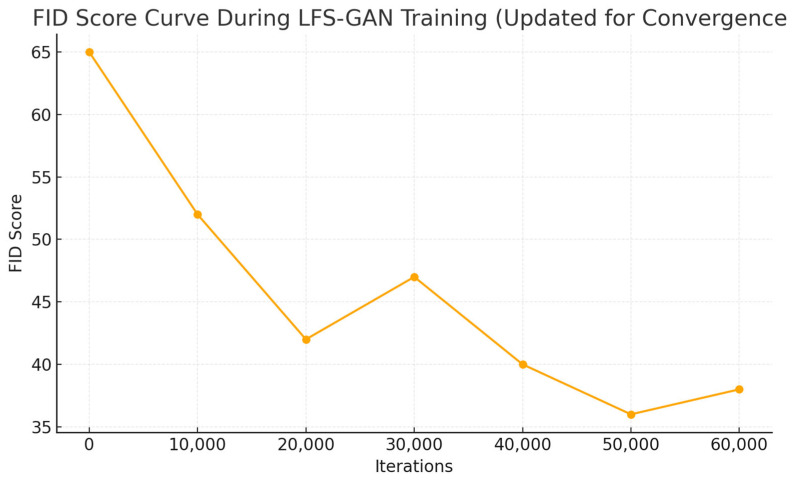
FID score curve during LFS-GAN training.

To comprehensively evaluate image quality, the Fréchet Inception Distance (FID) score was used. FID was recorded every 5000 iterations, covering the entire training process from iteration 0 to 60,000. The resulting trend is illustrated in Figure 20.

As shown in Figure 20, the FID score drops sharply from 64.2 to 43.5 during the initial 0–10,000 iterations, indicating rapid quality improvement. Around iteration 15,000, the FID slightly rebounds to 47.1, reflecting a brief degradation due to adversarial pressure affecting the generator. As training progresses, the model regains stability: FID gradually decreases from 40.8 at iteration 20,000 to 33.2 at iteration 35,000. During iterations 40,000 to 60,000, the decline becomes smoother, with FID values dropping from 28.5 to approximately 21.2—suggesting the model has largely converged to an optimal state, and the generated images closely resemble real images in visual quality.

### 4.2. Two-Dimensional Defect Detection

**Figure 21:**
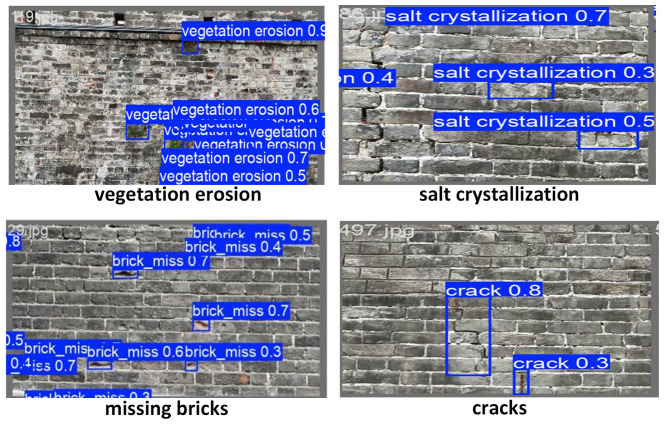
Detection results of YOLOv12.

For images of the ancient city wall collected by a UAV, the YOLOv12 model successfully detected the four typical defect types: missing bricks, cracks, salt crystallization, and vegetation erosion. As shown in Figure 21, YOLOv12 produced no obvious false positives or missed detections in the central and lower areas, indicating that its discrimination mechanism adapts well to defect variations across different levels of grayscale and texture density on the wall surface.

Even in areas where the defects of missing bricks, cracks, vegetation coverage, or salt crystallization were partially occluded by vegetation or blurred due to weathering, the model was still able to accurately identify them. This can be attributed to the structured attention module, which demonstrates a strong spatial edge response capability.

Table 6 presents the model’s detection performance across the four typical types of defects found in ancient city walls: cracks, salt crystallization, missing bricks, and vegetation erosion.

From the perspective of precision, all four defect types achieved scores above 0.83, indicating strong recognition capability and low false-positive rates. Among them, missing bricks (0.841) and vegetation erosion (0.844) exhibited the highest precision, likely due to their more prominent geometric or color-contrast features, which are easier for the model to detect.

In terms of recall, vegetation erosion (0.758) and cracks (0.752) outperformed the other categories, suggesting that the model has good coverage for these types and is less likely to miss instances. In contrast, salt crystallization had a slightly lower recall (0.739), possibly due to its blurry edges and texture similarity with the background, which increase detection difficulty.

Looking at the mAP metric, which reflects overall detection capability:

1. Vegetation erosion achieved the highest mAP at 0.834, indicating the model learns best from features with clear structural variation and strong contrast;
2. Cracks followed closely with an mAP of 0.832, showing the model’s effective extraction of fine-grained texture features;
3. Missing bricks and salt crystallization scored slightly lower at 0.819 and 0.827, respectively, but still reflect strong generalization performance across varying defect types.

Overall, the maximum mAP difference among the four defect categories was less than 0.015, demonstrating the model’s consistency and stability in handling diverse defect types. This balanced detection capability across different textures, structural scales, and boundary patterns forms a solid foundation for deploying the system in real-world scenarios where a wide range of defects must be accurately identified.

### 4.3. Depth Estimation

**Figure 22:**
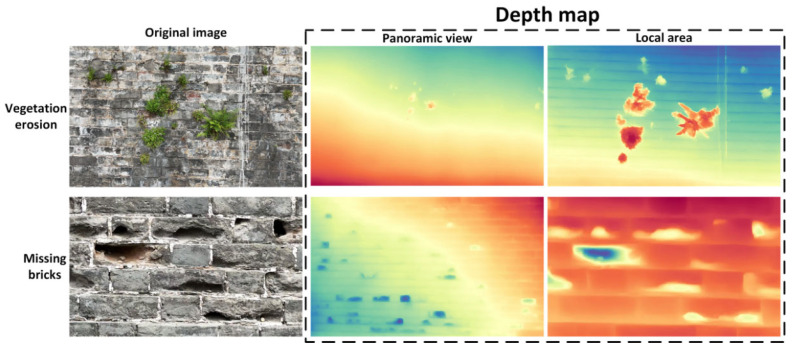
Depth estimation results by ZoeDepth for typical wall defects.

Figure 22 presents vegetation erosion and missing bricks under multiple views, including local and panoramic perspectives. Each view includes the original RGB image and the corresponding depth response map predicted by the model.

From the visualization results, the vegetation erosion regions are clearly represented by prominent red patches in the depth heatmaps, accurately corresponding to the vegetation clusters protruding from the wall in the original images. This confirms the model’s strong capability in recognizing structural occlusions.

The areas of missing bricks appear as distinct blue depressions in the depth heatmaps. Notably, in the local views, the model successfully reconstructs the depth discontinuities caused by brick loss, indicating high sensitivity to localized surface recessions.

To further quantitatively evaluate the quality of the system’s depth estimation under unsupervised conditions, Table 7 presents four commonly used no-reference depth map quality metrics.

Firstly, the depth entropy is 7.2326, indicating that the generated depth maps possess high informational complexity in their overall grayscale distribution. A higher entropy value reflects richer structural variation and detail, suggesting that the depth maps effectively capture fluctuations and disparities across different regions of the scene.

Secondly, the Sobel Edge Mean is 0.0185, which measures the sharpness of edge regions within the depth map. Although this value is relatively low, under conditions of limited training data and unsupervised learning, it still demonstrates the model’s ability to delineate wall outlines and defect boundaries—indicating a certain level of geometric boundary awareness guided by image features.

Furthermore, the depth standard deviation (Depth Std) is 45.7405, and the depth range reaches 247. These two indicators together reflect the model’s dynamic range and responsiveness in depth prediction. A larger standard deviation suggests that the output captures a broad range of depth levels, enabling differentiation of fine surface undulations and structural damage. A wider depth range indicates the model’s capacity to represent the 3D space from foreground to background, which aids in clearly separating defect regions from the background.

In summary, even without external supervision, the depth estimation module demonstrates strong geometric expressiveness and edge sensitivity. It maintains continuity in spatial structure while remaining responsive to local depth discontinuities caused by defects—offering a solid foundation for further 3D analysis and visualization in heritage preservation tasks.

### 4.4. Three-Dimensional Reconstruction

To evaluate the reconstruction quality and geometric representation capability of the 3D modeling module, Table 8 presents key no-reference quality metrics for 3D point clouds, including the point cloud density, normal vector consistency, and local boundary curvature response. Firstly, the average point cloud density reaches 148.7 points per unit volume, indicating that the system—powered by Gaussian Splatting—can generate a highly dense point cloud while ensuring full spatial coverage. This high-density representation is crucial for the 3D reconstruction of fine defects such as cracks and missing bricks, significantly enhancing the spatial resolution and accuracy of subsequent analysis. Secondly, the standard deviation of the point cloud density is 37.2, reflecting some variability in spatial distribution. However, this fluctuation remains within a controllable range, with no significant signs of density fragmentation. This suggests that the model adopts a relatively balanced sampling strategy across regions with varying textures, contributing to improved structural consistency in the overall reconstruction. Further analysis shows that the normal vector consistency reaches 0.9642, indicating a high degree of alignment in surface normals between point-cloud segments and smooth continuity across the reconstructed surface geometry.

Lastly, the local boundary curvature response is 0.038, demonstrating the system’s capability to effectively capture surface curvature variations. This is especially important for identifying geometric discontinuities at defect edges—such as crack corners or missing brick boundaries—thus supporting geometric segmentation and damage-level classification.

In conclusion, the Gaussian Splatting-based 3D reconstruction module in this system exhibits strong performance in terms of density, surface continuity, and boundary structure sensitivity. It provides robust spatial geometric perception and reasoning capabilities, forming a solid foundation for 3D visualization and heritage defect analysis.

**Figure 23:**
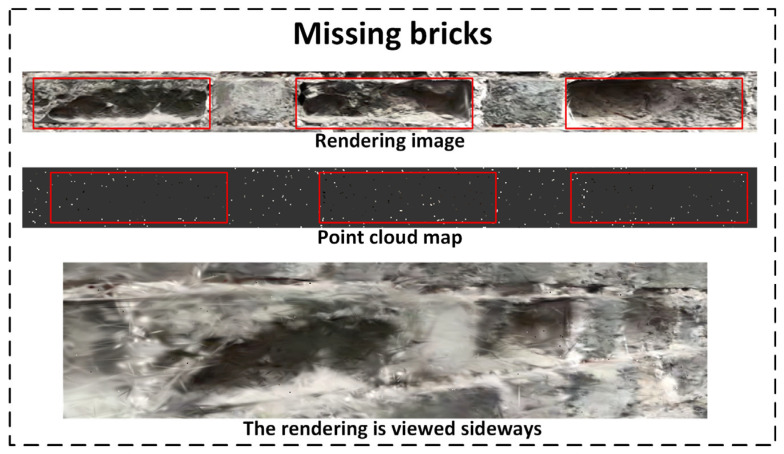
Sliced point cloud of missing brick area.

In Figure 23, regions with missing bricks—due to material loss and surface recession—exhibit significantly sparser point-cloud distributions, with noticeable “voids” or low-density zones. Compared to the intact wall sections, these areas show a clear drop in the Gaussian point density, forming discontinuities that facilitate accurate localization and identification. This “void” pattern serves as a key structural cue for identifying brick loss, contributing to the detection and restoration assessment of ancient wall defects.

It is worth noting that the point-cloud data not only enable the spatial localization and visualization of missing brick areas on ancient city walls but also hold significant potential for further quantitative analysis. By integrating high-precision point-cloud data with the actual dimensional information of the wall, the system can estimate the volume of each missing brick area, allowing for accurate calculation of the total volume loss. In addition, by identifying the number and spatial distribution of missing brick regions, the system can derive the overall quantity and cumulative volume of damage. These quantitative results provide crucial support for developing scientifically grounded restoration plans and help accurately assess the material requirements for repair projects. This, in turn, facilitates precise management and allocation of restoration resources. Therefore, point-cloud data not only enhance the spatial accuracy of defect detection but also offer a more scientific and practical technical foundation for informed decision-making and execution in ancient wall restoration efforts.

**Figure 24:**
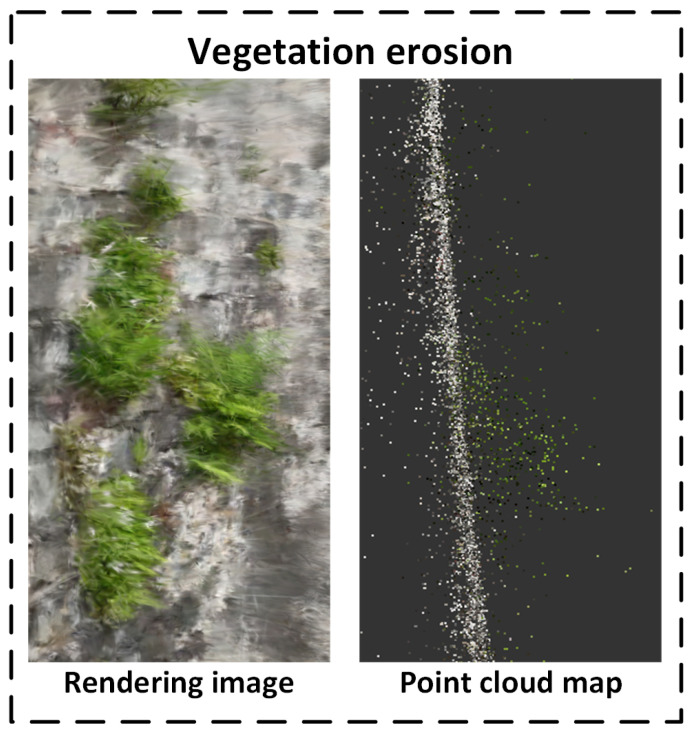
Sliced point cloud of vegetation erosion.

As shown in Figure 24, the vegetation erosion areas in the sliced point cloud reveal significant depth layer variation. Due to outward plant growth, these regions appear as 3D forward protrusions, with dense extensions along the depth axis. Furthermore, the rich texture and color variation in vegetation leads to high-density green point clouds in the rendering, which—when combined with depth protrusions—enable reliable discrimination from flat wall surfaces. This combined representation of depth protrusion and color texture provides robust data support for vegetation-related degradation assessment and intervention.

Furthermore, by combining the actual dimensions of the ancient city wall with the point-cloud model, it is possible to quantitatively estimate both the volume and surface area of vegetation-covered regions. This provides valuable data for assessing the potential structural impact of vegetation on the wall and for formulating targeted vegetation removal and restoration plans. In addition, continuous monitoring and comparison of point-cloud data over time enables dynamic tracking of vegetation growth and spread, offering a scientific basis for vegetation intervention and long-term wall preservation strategies. Therefore, point-cloud data not only improve the spatial accuracy of detecting vegetation erosion defects but also play a critical role in the ecological management of heritage sites and the rational allocation of restoration resources—delivering robust technical support for both conservation and decision-making.

**Figure 25:**
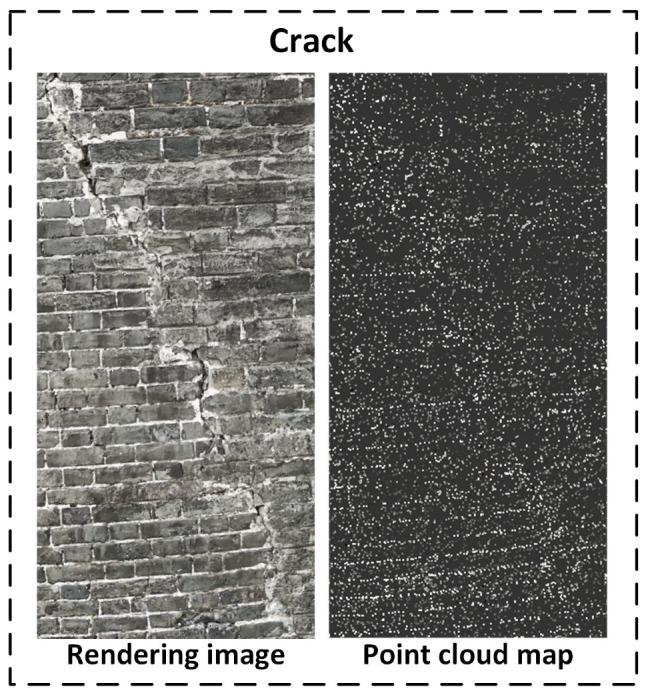
Sliced point cloud of crack defect.

As depicted in Figure 25, cracks are not represented by completely missing points. Instead, the surrounding regions exhibit locally concentrated point clouds, caused by geometric discontinuities and texture gradients near crack boundaries. The Gaussian points tend to cluster along the edges of the cracks, indirectly depicting the crack trajectory and morphology. Though the cracks may be too narrow to create voids, the resulting density anomalies in adjacent regions become valuable indicators of crack presence, especially when combined with texture information.

In addition, point-cloud data can be used to compare crack developments across different time periods, helping determine whether the cracks are actively progressing. This provides crucial data support for early warning systems and scientifically informed intervention strategies. Quantitative crack information can also be used to assess the impact of cracks on the structural integrity of the ancient city wall. It further supports the formulation of targeted reinforcement and repair plans, enabling accurate estimation of material requirements and the rational allocation of resources. Therefore, point-cloud reconstruction not only enhances the visualization and analytical precision of crack defects but also offers robust technical support for structural health monitoring and evidence-based restoration of ancient city walls.

**Figure 26:**
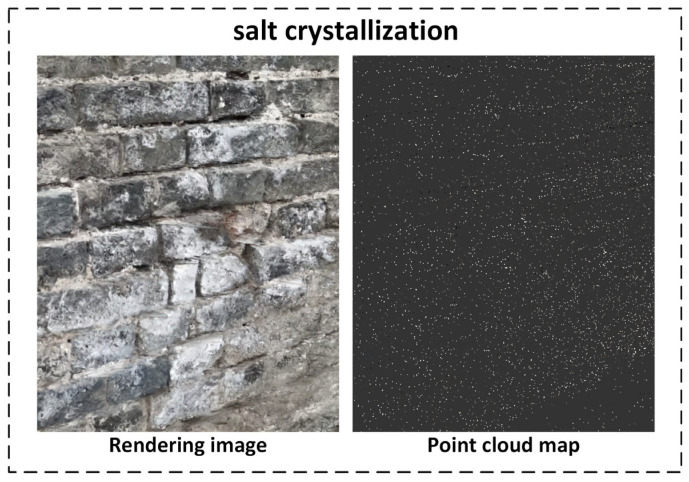
Sliced point cloud of salt crystallization defect.

Figure 26 demonstrates the characteristics of salt crystallization in the 3D reconstruction. Unlike missing bricks or cracks—which trigger noticeable geometric changes—salt crystallization manifests primarily as fine surface deposits or color shifts, lacking prominent depth or structural contrast. As a result, the point cloud exhibits uniform density, with no distinct clusters or gaps, making it difficult to detect this defect type based solely on geometric information. Therefore, YOLOv12-based 2D object detection is employed for effective recognition of salt crystallization.

In conclusion, the Gaussian Splatting-based reconstruction method enables multi-perspective visual expression of structural damage on ancient city walls. Through detailed analyses of the four major defect types, this approach offers intuitive and high-fidelity 3D data, serving as a robust foundation for the digital preservation, analysis, and restoration of historical masonry heritage.

## 5. Conclusions

This study presents a comprehensive defect-detection system for ancient city walls, integrating multi-stage processing modules including image enhancement, 2D detection, depth estimation, and 3D modeling. The system enables intelligent identification and visual representation of structural deterioration. It addresses common challenges in ancient wall image acquisition—such as motion blur, low illumination, and limited sample size—by proposing generalized and adaptable solutions. The overall system demonstrates strong adaptability and promising application potential.

The main contributions and research conclusions of this work are as follows:

1. A multi-strategy image enhancement pipeline combining Restormer, SG-LLIE, and LFS-GAN is introduced to address issues related to motion blur, low-light conditions, and limited data availability, ensuring robust input quality for downstream detection tasks.
2. A dual-stage detection framework based on YOLOv12 and ZoeDepth is developed, which fuses 2D detection results with relative depth estimation. This enhances spatial perception and improves the overall robustness and accuracy of the defect recognition system.
3. By leveraging Gaussian Splatting-based point-cloud reconstruction, the system achieves multi-view 3D visualization and geometric analysis of wall defects, offering a novel and effective approach for the digital conservation and assessment of historic masonry structures.

When the three image-processing modules—deblurring, illumination enhancement, and data augmentation—were used in combination, the system achieved optimal detection performance (mAP = 0.839, precision = 0.844, and recall = 0.765). This demonstrates the synergistic effect of the integrated enhancement strategy in boosting the model’s perception capability, providing high-quality input for subsequent defect detection.

In the detection module, the YOLOv12 model exhibited stable average performance across four typical defect categories. The average precision for all four defect types reached 85%, with an overall mAP of approximately 0.828, indicating strong accuracy and generalization capability in distinguishing between different defect types. During the depth estimation phase, the system produced high-quality depth maps under unsupervised conditions. Specifically, depth entropy reached 7.2326, suggesting that the depth maps contain a high level of structural information; depth standard deviation (Depth Std) was 45.7405, reflecting the model’s strong ability to distinguish depth differences across various defect areas. In the point-cloud modeling stage, the average point cloud density reached 148.7 points/unit volume, and the normal vector consistency was 0.9642—both indicating the high geometric continuity and effective structural reconstruction capabilities of the 3D model. From a technological development perspective, there remains room for further optimization and expansion. Advancing model lightweighting and embedded deployment will improve the system’s practical field applicability, and incorporating multi-temporal image or point-cloud data for the same locations will enable longitudinal analysis of defect progression over time, supporting sustained monitoring and dynamic tracking.

In summary, the ancient city wall defect-detection system developed in this study demonstrates strong theoretical design, effective algorithm integration, and successful experimental validation. It exhibits robust adaptability and practical application value, offering a viable technical pathway for the intelligent preservation of architectural heritage.

## Figures and Tables

**Table 1:** Comparison of methods at each stage in the ancient city wall defect-detection pipeline.

Detection Stage	Method	Function	Advantage	Applicable Defect
Initial screening	YOLOv12	Identification of image cracks, salting out, vegetation erosion, and missing bricks	Fast reasoning and high detection rate	Cracks, salt crystallization, missing bricks, vegetation erosion
Supplementary identification	Depth Estimation	Abnormal fluctuation trend detection	The model is lightweight and has an abnormal response structure	Missing bricks, vegetation erosion
Precise modeling	Gaussian Splatting	Point-cloud restoration	3D visual representation of the defects	Cracks, missing bricks, vegetation erosion

**Table 2:** Comparison of image quality metrics before and after deblurring using the Restormer model.

Indicator Name	Numerical Value of Blurred Image	Deblurred Image Values	Improve or Compare
Laplacian Variance	1191.62	2225.72	86.8%
Edge Gradient Mean	101.81	142.26	39.7%
SSIM ↑	0.1659	0.834	402.7%
PSNR ↑	14.10 dB	24.66 dB	74.9%

↑: The higher this value is, the better.

**Table 3:** Performance comparison between SG-LLIE and Retinex in illumination enhancement.

Team	PSNR ↑	SSIM ↑	LPIPS ↓	NIQE ↓
SG-LLIE	25.898	0.851	0.127	11.8284
Retinex	22.408	0.65	0.202	4.786

↑: The higher this value is, the better. ↓: The higher this value is, the better.

**Table 4:** Ablation study results of defect detection performance under different image-processing module configurations.

Configuration	Deblurring	Illumination Enhancement	LFS-GAN Augmentation	mAP	Precision	Recall
None	×	×	×	0.751	0.733	0.689
Only Deblurring	√	×	×	0.804	0.827	0.745
Only Illumination	×	√	×	0.792	0.817	0.712
Only LFS-GAN	×	×	√	0.811	0.817	0.728
All Modules	√	√	√	0.839	0.844	0.765

**Table 5:** Training configuration for LFS-GAN model.

Project	Parameter Value
GPU model	NVIDIA RTX 4090 24 GB
Learning rate (generator)	1 × 10^−4^
Learning rate (discriminator)	4 × 10^−4^
Batch size	16
Input image resolution	256 × 256
Optimizer	Adam (β1 = 0.5, β2 = 0.999)
Pattern diversity loss weight	0.3
Model preservation period	Every 5000 rounds
Total training rounds	60,000

**Table 6:** Detection metrics for different defect types.

Type	Precision	Recall	mAP
Cracks	0.839	0.752	0.832
Salt Crystallization	0.838	0.739	0.827
Missing Bricks	0.841	0.740	0.819
Vegetation Erosion	0.844	0.758	0.834

**Table 7:** No-reference evaluation metrics for depth estimation.

Indicator Name	Depth Entropy	Sobel Edge Mean	Depth Std	Depth Range
Numerical value	7.2326	0.0185	45.7405	247

**Table 8:** No-reference 3D point-cloud reconstruction quality evaluation metrics.

Evaluation Index	Average Point Cloud Density	Standard Deviation of Point Cloud Density	Normal Vector Consistency	Local Boundary Curvature Response
Simulated numerical value	148.7	37.2	0.9642	0.038

## Data Availability

The original data presented in the study are openly available.

## References

[1] Guo Z., Qi Q., Zhang S., Chen W., Wu C., Wu H. (2023). Study on the characterization of differential weathering feature based on surface roughness theory and 3D laser scanning: A case study of the Suoyang Ancient City. J. Cult. Herit..

[2] Chen Z., Li Z., Li M., Zhao X., Zhang Y. (2024). Analysis of differential weathering patterns and causes on the top of hollow enemy towers’ facades of the Great Wall—A case study of section in Haigang District, Qinhuangdao City. J. Asian Archit. Build. Eng..

[3] Ye L., Shao J., Sun P., Lv W., Fang M., Jin Y. (2022). Analysis and influence of rainfall infiltration of ancient city wall based on in-situ monitoring: A case study of The XingChun gate Lishui Prefecture. Métodos Numér. Cálc. Diseño Ing..

[4] Wang Y., Song J., Zhang J., Huang Y., Yang S. (2025). Analysis of Rammed Earth Wall Erosion in Traditional Village Dwellings in Zhuhai City. Coatings.

[5] Shang R., Sun F., Lu H., Han P., Fu J., Liu W. (2024). Impact Mechanism of Vegetation Slope Surface on the Runoff and Sediment Producing Procedure of Jinyang Ancient City in Different Periods. Environ. Eng. Manag. J..

[6] Gao S., Tao L., Chen F., Zhou X., Shi P., Yao X., Zhu M. (2024). Extraction of deterioration and analysis of vegetation impact effects on the south palace wall of Weiyang Palace. Herit. Sci..

[7] Ma S., Chun Q., Zhang C., Li D., Zhai F., Yuan Y. (2025). Automatic damage detection and localization of ancient city walls—A case study of the Great Wall. npj Herit. Sci..

[8] Wu J., Shi Y., Wang H., Wen Y., Du Y. (2023). Surface defect detection of Nanjing City Wall based on UAV oblique photogrammetry and TLS. Remote Sens..

[9] Resende M.M., Gambare E.B., Silva L.A., Cordeiro Y.S., Almeida E., Salvador R.P. (2022). Infrared thermal imaging to inspect pathologies on façades of historical buildings: A case study on the Municipal Market of São Paulo, Brazil. Case Stud. Constr. Mater..

[10] Brooke C. (2018). Thermal imaging for the archaeological investigation of historic buildings. Remote Sens..

[11] Adamopoulos E., Volinia M., Girotto M., Rinaudo F. (2020). Three-dimensional thermal mapping from IRT images for rapid architectural heritage NDT. Buildings.

[12] Ludeno G., Cavalagli N., Ubertini F., Soldovieri F., Catapano I. (2020). On the combined use of ground penetrating radar and crack meter sensors for structural monitoring: Application to the historical Consoli Palace in Gubbio, Italy. Surv. Geophys..

[13] Mohammed M., Thabit J.M., Firas H. (2023). Electrical resistivity tomography and ground-penetrating radar methods to detect archaeological walls of Babylonian houses near Ishtar temple, ancient Babylon city, Iraq. Geophys. Prospect..

[14] Guo J., Ma J., García-Fernández Á.F., Zhang Y., Liang H. (2023). A survey on image enhancement for Low-light images. Heliyon.

[15] Kim W. (2022). Low-light image enhancement: A comparative review and prospects. IEEE Access.

[16] Perez-Zarate E., Ramos-Soto O., Liu C., Oliva D., Perez-Cisneros M. (2025). ALEN: A dual-approach for uniform and non-uniform low-light image enhancement. Multimed. Syst..

[17] Wang L., Zhao L., Zhong T., Wu C. (2024). Low-light image enhancement using generative adversarial networks. Sci. Rep..

[18] Liu Y., Liu Y., Pan J., Hui Y., Jia F., Chan R.H., Zeng T. (2024). Super-resolving Real-world Image Illumination Enhancement: A New Dataset and A Conditional Diffusion Model. arXiv.

[19] Zhang J., Li Z., Zhang J., Wang Y. (2025). Retinex-Based Self-Conditioned Diffusion Model for Low-Light Image Enhancement. Proceedings of the ICASSP 2025-2025 IEEE International Conference on Acoustics, Speech and Signal Processing (ICASSP).

[20] Wang S., Zhang S., Wu J., Tian Z., Chen W., Jin T., Xue M., Wang Z., Yu F.R., Leung V. (2025). CLIP-Optimized Multimodal Image Enhancement via ISP-CNN Fusion for Coal Mine IoVT under Uneven Illumination. arXiv.

[21] Turab M. (2025). A Comprehensive Survey on Image Signal Processing Approaches for Low-Illumination Image Enhancement. arXiv.

[22] Yuan Q., Shi Y., Li M. (2024). A review of computer vision-based crack detection methods in civil infrastructure: Progress and challenges. Remote Sens..

[23] Zhang J., Qian S., Tan C. (2022). Automated bridge surface crack detection and segmentation using computer vision-based deep learning model. Eng. Appl. Artif. Intell..

[24] Mirbod M., Shoar M. (2023). Intelligent concrete surface Cracks detection using computer vision, pattern recognition, and artificial neural networks. Procedia Comput. Sci..

[25] Chen W., Meng S., Wang X. (2024). Local and global context-enhanced lightweight CenterNet for PCB surface defect detection. Sensors.

[26] Liu H., Xu Y., Huang J., Bao Q., Wen Z. (2025). Surface defect detection based on machine learning and data augmentation. Proceedings of the 2024 IEEE 6th International Conference on Civil Aviation Safety and Information Technology (ICCASIT).

[27] Li Q., Xu X., Guan J., Yang H. (2024). The improvement of Faster-RCNN crack recognition model and parameters based on attention mechanism. Symmetry.

[28] Wang H., Wang Q., Zhang W., Zhai J., Yuan D., Tong J., Xie X., Zhou B., Tian H. (2025). A Deep Learning-Based Watershed Feature Fusion Approach for Tunnel Crack Segmentation in Complex Backgrounds. Materials.

[29] Ruzavina I., Theis L.S., Lemeer J., de Groen R., Ebeling L., Hulak A., Ali J., Tang G., Mockel R. (2025). SteelBlastQC: Shot-blasted Steel Surface Dataset with Interpretable Detection of Surface Defects. arXiv.

[30] Li R., Zhao L., Wei H., Hu G., Xu Y., Ouyang B., Tan J. (2025). Multi-defect type beam bridge dataset: GYU-DET. Sci. Data.

[31] Wang Q. (2025). Monocular line scan vision-based surface defect detection approach for highly reflective bearing balls. Opt. Lett..

[32] Zhao Z., Li T. (2025). Enhancing wind turbine blade damage detection with YOLO-Wind. Sci. Rep..

[33] Dou X., Xue C., Zhang G., Jiang Z. (2024). Internal thread defect detection system based on multi-vision. PLoS ONE.

[34] Zhang X., Huo L., Liu Y., Zhuang Z., Yang Y., Gou B. (2023). Research on 3D phenotypic reconstruction and micro-defect detection of green plum based on multi-view images. Forests.

[35] Wang Y., Sun W., Jin J., Kong Z., Yue X. (2023). MVGCN: Multi-view graph convolutional neural network for surface defect identification using three-dimensional point cloud. J. Manuf. Sci. Eng..

[36] Arav R., Wittich D., Rottensteiner F. (2025). Evaluating saliency scores in point clouds of natural environments by learning surface anomalies. ISPRS J. Photogramm. Remote Sens..

[37] Liu J., Mou S., Gaw N., Wang Y. (2025). Uni-3DAD: Gan-inversion aided universal 3D anomaly detection on model-free products. Expert Syst. Appl..

[38] Yang X., Sun J., Ma L., Zhou X., Lu W., Li S. (2024). Research on the Depth Image Reconstruction Algorithm Using the Two-Dimensional Kaniadakis Entropy Threshold. Sensors.

[39] Zhou K., Cao Y., Kim T., Zhao H., Dong H., Ting K.M., Zhu Y. (2024). RAD: A dataset and benchmark for real-life anomaly detection with robotic observations. arXiv.

[40] Deng P., Yao J., Li C., Wang S., Li X., Ojha V., He X., Matsumoto T. (2025). Unified Few-shot Crack Segmentation and its Precise 3D Automatic Measurement in Concrete Structures. arXiv.

[41] Wei W., Wei P., Liao Z., Qin J., Cheng X., Liu M., Zheng N. (2023). Semantic Consistency Reasoning for 3-D Object Detection in Point Clouds. IEEE Trans. Neural Netw. Learn. Syst..

[42] Chen B., Lv X., Zhao Y., Yu L. (2024). TPDC: Point Cloud Completion by Triangular Pyramid Features and Divide-and-Conquer in Complex Environments. IEEE Trans. Neural Netw. Learn. Syst..

[43] Crisan A., Pepe M., Costantino D., Herban S. (2024). From 3D point cloud to an intelligent model set for cultural heritage conservation. Heritage.

[44] Liu Y., Chen J. (2023). Research on the Conservation of Historical Buildings Based on Digital 3D Reconstruction. Procedia Comput. Sci..

[45] Yang S., Xu S., Huang W. (2022). 3D point cloud for cultural heritage: A scientometric survey. Remote Sens..

[46] Zhang K., Ren W., Luo W., Lai W.-S., Stenger B., Yang M.-H., Li H. (2022). Deep image deblurring: A survey. Int. J. Comput. Vis..

[47] Li C. (2022). A survey on image deblurring. arXiv.

[48] Yang S., Xiao W., Zhang M., Guo S., Zhao J., Shen F. (2022). Image data augmentation for deep learning: A survey. arXiv.

[49] Kumar T., Brennan R., Mileo A., Bendechache M. (2024). Image data augmentation approaches: A comprehensive survey and future directions. IEEE Access.

[50] Zamir S.W., Arora A., Khan S., Hayat M., Khan F.S., Yang M.-H. Restormer: Efficient transformer for high-resolution image restoration. Proceedings of the IEEE/CVF Conference on Computer Vision and Pattern Recognition.

[51] Bhatt D., Patel C., Talsania H., Patel J., Vaghela R., Pandya S., Modi K., Ghayvat H. (2021). CNN variants for computer vision: History, architecture, application, challenges and future scope. Electronics.

[52] Kattenborn T., Leitloff J., Schiefer F., Hinz S. (2021). Review on Convolutional Neural Networks (CNN) in vegetation remote sensing. ISPRS J. Photogramm. Remote Sens..

[53] Tang H., Zhu H., Fei L., Wang T., Cao Y., Xie C. (2023). Low-illumination image enhancement based on deep learning techniques: A brief review. Photonics.

[54] Dong W., Min Y., Zhou H., Chen J. Towards Scale-Aware Low-Light Enhancement via Structure-Guided Transformer Design. Proceedings of the Computer Vision and Pattern Recognition Conference.

[55] Wu Q., Chen Y., Meng J. (2020). DCGAN-based data augmentation for tomato leaf disease identification. IEEE Access.

[56] Chu C., Zhmoginov A., Sandler M. (2017). Cyclegan, a master of steganography. arXiv.

[57] Karras T., Laine S., Aittala M., Hellsten J., Lehtinen J., Aila T. Analyzing and improving the image quality of stylegan. Proceedings of the IEEE/CVF Conference on Computer Vision and Pattern Recognition.

[58] Seo J., Kang J.-S., Park G.-M. Lfs-gan: Lifelong few-shot image generation. Proceedings of the IEEE/CVF International Conference on Computer Vision.

[59] Kossale Y., Airaj M., Darouichi A. (2022). Mode collapse in generative adversarial networks: An overview. Proceedings of the 2022 8th International Conference on Optimization and Applications (ICOA).

[60] Dong Y., Xia C., Yang J., Cao Y., Cao Y., Li X. (2021). Spatio-temporal 3-D residual networks for simultaneous detection and depth estimation of CFRP subsurface defects in lock-in thermography. IEEE Trans. Ind. Inform..

[61] Azizinasab B., Hasanzadeh R.P.R., Hedayatrasa S., Kersemans M. (2021). Defect detection and depth estimation in CFRP through phase of transient response of flash thermography. IEEE Trans. Ind. Inform..

[62] Voronin V., Gapon N., Zhdanova M., Tokareva O., Khamidullin I., Semenishchev E. (2023). Deep learning-based depth map defect removal for industrial applications. Proceedings of the Optical Metrology and Inspection for Industrial Applications X.

[63] Tian Y., Ye Q., Doermann D. (2025). Yolov12: Attention-centric real-time object detectors. arXiv.

[64] Soni A., Rai A. (2024). YOLO for Medical Object Detection (2018–2024). Proceedings of the 2024 IEEE 3rd International Conference on Electrical Power and Energy Systems (ICEPES).

[65] Mertan A., Duff D.J., Unal G. (2022). Single image depth estimation: An overview. Digit. Signal Process..

[66] Bhat S.F., Birkl R., Wofk D., Wonka P., Müller M. (2023). Zoedepth: Zero-shot transfer by combining relative and metric depth. arXiv.

[67] Kerbl B., Kopanas G., Leimkühler T., Drettakis G. (2023). 3d gaussian splatting for real-time radiance field rendering. ACM Trans. Graph..

[68] Wang Z., Zhou Q., Shuang Y. (2020). Three-dimensional reconstruction with single-shot structured light dot pattern and analytic solutions. Measurement.

[69] Nguyen T.T., Slaughter D.C., Max N., Maloof J.N., Sinha N. (2015). Structured light-based 3D reconstruction system for plants. Sensors.

[70] Cui B., Tao W., Zhao H. (2021). High-precision 3D reconstruction for small-to-medium-sized objects utilizing line-structured light scanning: A review. Remote Sens..

[71] Kühner T., Kümmerle J. (2020). Large-scale volumetric scene reconstruction using lidar. Proceedings of the 2020 IEEE International Conference on Robotics and Automation (ICRA).

[72] Huang J., Stoter J., Peters R., Nan L. (2022). City3D: Large-scale building reconstruction from airborne LiDAR point clouds. Remote Sens..

[73] Chen Y., Wen C., Liu W., He W. (2023). A depth iterative illumination estimation network for low-light image enhancement based on retinex theory. Sci. Rep..

